# Characterization of the mitochondrial genomes of three powdery mildew pathogens reveals remarkable variation in size and nucleotide composition

**DOI:** 10.1099/mgen.0.000720

**Published:** 2021-12-10

**Authors:** Alex Z. Zaccaron, Ioannis Stergiopoulos

**Affiliations:** ^1^​ Department of Plant Pathology, University of California Davis, Davis, CA, USA

**Keywords:** cytochrome b, Erysiphales, fungicide resistance, homing endonuclease, horizontal transfer, reverse transcriptase

## Abstract

Powdery mildews comprise a large group of economically important phytopathogenic fungi. However, limited information exists on their mitochondrial genomes. Here, we assembled and compared the mitochondrial genomes of the powdery mildew pathogens *Blumeria graminis* f. sp. *tritici*, *Erysiphe pisi*, and *Golovinomyces cichoracearum*. Included in the comparative analysis was also the mitochondrial genome of *Erysiphe necator* that was previously analysed. The mitochondrial genomes of the four Erysiphales exhibit a similar gene content and organization but a large variation in size, with sizes ranging from 109800 bp in *B. graminis* f. sp. *tritici* to 332165 bp in *G. cichoracearum*, which is the largest mitochondrial genome of a fungal pathogen reported to date. Further comparative analysis revealed an unusual bimodal GC distribution in the mitochondrial genomes of *B. graminis* f. sp. *tritici* and *G. cichoracearum* that was not previously observed in fungi. The cytochrome *b* (*cob*) genes of *E. necator, E. pisi*, and *G. cichoracearum* were also exceptionally rich in introns, which in turn harboured rare open reading frames encoding reverse transcriptases that were likely acquired horizontally. *Golovinomyces cichoracearum* had also the longest *cob* gene (45 kb) among 703 fungal *cob* genes analysed. Collectively, these results provide novel insights into the organization of mitochondrial genomes of powdery mildew pathogens and represent valuable resources for population genetics and evolutionary studies.

## Data Summary

The assembled mitochondrial genomes of *B. graminis* f. sp. *tritici*, *E. pisi*, and *G. cichoracearum* have been submitted to GenBank under the accession numbers MT880591, MT880589, and MT880590, respectively. Accession numbers of all other mitochondrial genomes and cytochrome *b* genes utilized in the comparative analyses are listed in Tables S1 and S8 (available in the online version of this article), respectively. Scripts utilized in this study were deposited in a public GitHub repository available at https://github.com/alexzaccaron/2021_pms_mt. The authors confirm all supporting data, code and protocols have been provided within the article or through supplementary data files.

**Impact Statement**

Mitochondria are increasingly the focus of many studies, mainly due to their importance as regulators of key cellular functions and the significance of mitochondrial DNA mutations in evolutionary biology and disease. In this study we assembled and analysed the mitochondrial genomes of *Blumeria graminis* f. sp. *tritici*, *Erysiphe pisi,* and *Golovinomyces cichoracearum*, three economically important powdery mildew pathogens, and further compared them with the mitochondrial genome of the grape powdery mildew fungus *Erysiphe necator* that was already available. Our analysis showed that powdery mildew fungi have mitochondrial genomes that vary remarkably in size, with *G. cichoracearum* possessing the largest mitochondrial genome reported to date for a plant pathogenic fungus. Further comparative analyses with other fungi revealed additional idiosyncratic features, including an atypical bimodal GC content distribution in the mitochondrial genomes of *B. graminis* f. sp. *tritici* and *G. cichoracearum* that was not observed in other fungi. Moreover, the cytochrome *b* genes of powdery mildew pathogens were among the largest and richest in introns among fungi, and harboured rare intronic ORFs encoding reverse transcriptases that were likely acquired via horizontal transfer. Taken together, our findings provide novel insights into the architecture and evolution of the mitochondrial genomes of powdery mildew fungi, and create a basis for future population genetic and evolutionary studies of these pathogens.

## Introduction

Mitochondria are organelles that perform critical functions in most eukaryotic cells. They are responsible for energy production through oxidative phosphorylation, and are further involved in iron metabolism, ageing, and programmed cell death [1, 2]. According to the endosymbiotic hypothesis, mitochondria are the descendants of an alpha-proteobacterium that was engulfed by an ancient eukaryotic cell, thus giving rise to a symbiotic relationship [3]. This hypothesis is supported by the fact that mitochondria have their own, typically circular, genomes. Studies suggest that most of the ancient endosymbiont genes migrated to the nuclear genome or were lost throughout evolution, resulting in a substantial size reduction of the mitochondrial (mt) genome [4]. Nonetheless, remarkable mt genome size variation has been observed, particularly among fungi in which it ranges from 12 kb in *Rozella allomycis* [5] to 531 kb in *Morchella crassipes* [6].

Size variation among fungal mt genomes can often be explained by the number and size of introns present in their genes [7], with smaller mt genomes typically containing no or few introns and larger mt genomes being enriched in mt introns [8–10]. Based on their secondary RNA structures and splicing mechanism, mt introns are typically classified into group I and group II introns, with group I introns further being classified into seven subgroups, i.e. IA, IA3, IB, IC1, IC2, ID, and I derived (I*) [11, 12]. Fungal mt introns also frequently host open reading frames (ORFs) encoding homing endonucleases (HEs) of the LAGLIDADG or GIY-YIG families, or reverse transcriptases (RTs), with the former most frequently found in group I introns and the latter in group II introns [13, 14]. HEs enable intron self-splicing and transposition to an intronless cognate allele by cleaving DNA at specific target sequences of 13 to 40 bp long. This, in turn, activates the double-strand break repair mechanism of the cell that converts the intron^−^ to an intron^+^ allele, by using the intron^+^ allele carrying the HE gene as template [15]. In contrast, the mobility of group II introns carrying RT-coding ORFs is similar to that of retroelements in which RNA reverse splice into a DNA target, followed by reverse transcription [14].

Despite their differences in size and intron content, the majority of fungal mt genomes typically contain a set of 14 core genes encoding proteins involved in the electron transport chain (ETC) and oxidative phosphorylation [8, 16]. These include *atp6*, *atp8,* and *atp9* that code for subunits 6, 8, and 9 of the ATP-synthase complex; *nad1*, *nad2*, *nad3*, *nad4*, *nad4L*, *nad5,* and *nad6* that code for seven Type I NADH dehydrogenases subunits of complex I; *cob* that codes for cytochrome b, one of the three catalytic subunits of complex III; and finally *cox1*, *cox2,* and *cox3* that code for the cytochrome c oxidase subunits 1, 2, and 3, respectively of complex IV [16]. In addition to protein-coding genes, fungal mt genomes also contain a set of mt-tRNAs as well as two genes, *rns* and *rnl*, encoding the small and large ribosomal subunits, respectively [8, 16].

Mt genomes are an important genomic resource for studies in population genetics and evolutionary genomics. For phytopathogenic fungi, they are also of particular significance because mutations in mt proteins are often associated with resistance to fungicides that block the electron transport chain (ETC) and the production of energy in cells. The quinone outside inhibitor (QoI) fungicides, for instance, inhibit mt respiration by binding to the outer quinol-oxidation (Q_o_) site of mt complex III, thus halting the production of ATP [17]. Resistance to QoIs in fungi has been predominately associated with point mutations in the cytochrome *b* (*cob*) gene, with the most common mutations leading to the amino acid (aa) substitutions p.F129L, p.G137R, and p.G143A [18]. Of these, the p.G143A mutation has been shown to confer high levels of resistance to QoIs, often leading to the collapse of pathogen control in the fields. However, the formation of this amino acid substitution is blocked in some species by the presence of an intron at codon 143, as on the nucleotide level the substitution leads to the disruption of the intron splicing site that prevents intron splicing and leads to a frameshift. Thus, the presence or absence of this intron in cytochrome *b* is sometimes used as a predictor of resistance development to QoIs by the p.G143A mutation, although it is reported that in some species, such as *Botrytis cinerea* [19] and *Monilinia* spp. [20], different isolates carry different alleles of the cytochrome *b* gene that differ in the presence or absence of this intron.

Powdery mildew (PM) fungi represent a large and diverse group of obligate biotrophic Ascomycetes (Leotiomycetes, Erysiphales) that cause diseases in a wide range of monocots and dicots. The Erysiphales consists of approximately 900 species from 18 genera that infect nearly 10000 species of angiosperms, including fruit trees, cereals, vegetables, and flowering plants, which highlights the economic importance of these pathogens [21]. Notably, the wheat PM fungus *Blumeria graminis* f. sp. *tritici* was recently ranked number eight among pests and pathogens that are causing the highest yield losses in wheat worldwide [22]. In contrast to the monocot-adapted PM fungus *B. graminis* f. sp. *tritici* that only infects wheat, *Golovinomyces cichoracearum, Erysiphe pisi,* and *Erysiphe necator* are dicot-adapted fungi. Specifically, *Erysiphe pisi* is the causal agent of pea PM. It can colonize almost all parts of the plant and under severe infections it may induce yield losses of up to 50% [23, 24]. *Erysiphe necator* causes PM on grapes. It is distributed globally in grape producing areas and is considered the major disease threat to grape fruit production and quality [25]. Finally, *Golovinomyces cichoracearum* is another dicot-adapted PM pathogen that has a broad host range that encompasses many plant species within members of the Asteraceae and Cucurbitaceae families, including important economic crops like lettuce, potato, melon, and several others [26, 27].

Despite the importance of PM fungi, genomic resources, including of mt genomes, for these plant pathogens are still scarce. Recently, the mt genome of *E. necator* was annotated and analysed [28]. It revealed an atypical mt gene organization that differs from other fungal species and has one of the highest among fungi number of mt introns. To further gain an insight into the mt genomes of PM fungi, here we assembled and annotated the complete mt genomes of *B. graminis* f. sp. *tritici*, *E. pisi,* and *G. cichoracearum*. We next performed a comparative genomic analysis among the four PM fungi. The results herein provide novel insights into mt genome organization within Erysiphales and constitute valuable genomic resources for population genetic studies of PM pathogens.

## Methods

### Assembly of mt genomes

To assemble the mitochondrial (mt) genome of *B. graminis* f. sp. *tritici* isolate 96224, reads were obtained from NCBI (SRR7642212) and trimmed with fastp v0.20 [29]. Reads representing the mt genome of *B. graminis* f. sp. *tritici* were extracted based on exact *k*-mer matching (*k*=29) performed with the *bbduk.sh* script of the BBMap v38-60 software package [30]. A scaffold (UNSH01000099.1) containing the mt genome of *B. graminis* f. sp. *hordei* isolate RACE1 [31] was utilized as bait to extract the reads. Extracted reads were processed with the *bbnorm.sh* script of BBMap to normalize the coverage to 100×. The resulting reads (total of 179 166 paired-end reads) were then assembled with SPAdes v3.14 [32] with the flag *isolate* enabled and *k*-mer values of 21, 33, 55, and 77. With these parameters, SPAdes assembled a contig of 109874 bp, which was further polished with Pilon v1.23 [33]. To assemble the mt genome of *E. pisi* isolate Palampur-1, PacBio reads were obtained from NCBI (SRR11059780). PacBio reads were processed with the *removesmartbell.sh* script of the BBMap v38-60 software package to remove remaining SMRTbell adapters and then mapped to the mt genome of *E. necator* [28] with minimap2 v2.16 [34] with parameters to map PacBio reads (option *map-pb*) and output the alignment in SAM format (option *-a*). The output of minimap2 was converted to BAM format and reads that successfully mapped were extracted with SAMtools v1.9. From the extracted reads, the 27 238 longest reads estimated to cover the mt genome 100 times were selected and then assembled with Canu v1.9 [35] using parameters *genomeSize=200k* and *corOutCoverage=60*. Canu assembled a 215191 bp contig, which was further polished with Flye v2.7 [36] based on three polishing rounds. To assemble the mt genome of *G. cichoracearum* isolate UCSC1, PacBio reads were downloaded from NCBI (SRR6829655) and processed with the *removesmartbell.sh* script of the BBMap v38-60 software package. First, coding sequences of mt genes from *E. necator* [28] were queried with BLASTn (e-value <1e-10) against the genome assembly of *G. magnicellulatus* isolate FPH2017-1 [37]. Three contigs (VCMJ01000001.1, VCMJ01000002.1, and VCMJ01000079.1) were identified to represent fragments of the mt genome of *G. magnicellulatus*. Subsequently, the PacBio reads of *G. cichoracearum* were mapped to these three contigs with minimap2 and successfully mapped reads were extracted with SAMtools v1.9. From the mapped reads, the longest 4912 reads were estimated to cover the mt genome 100 times and were then assembled with Canu v1.9 with parameters *genomeSize=350k* and *corOutCoverage=60*. Canu assembled a 358768 bp contig, which was further polished with Flye v2.7 based on three polishing rounds. A final round of polishing was carried out with Pilon v1.23, by utilizing Illumina reads of *G. cichoracearum* isolate UCSC1 (SRR4017398) mapped to the assembled mt genome with BWA-MEM v0.7.17 [38]. Self BLASTn searches were performed to identify overlapping ends of the assembled contigs, suggesting circularity. For *B. graminis* f. sp. *tritici*, the first and last 77 bp of the assembled contig were identical. For *E. pisi* and *G. cichoracearum* overlapping ends of 26.7 kb and 26.6 kb were identified to overlap almost perfectly, with 99.6 and 99.8% identity, respectively. One of the overlapping ends were removed and the contigs were rotated so that the first position was the start coordinate of the *cox1* gene. The workflow of how the genomes were assembled is shown in Fig. S1.

### Annotation of mt genomes

Assembled mt genomes were initially annotated with the MFannot webserver, using the genetic code 4 (Mould, Protozoan and Coelenterate Mt Code) [39]. To validate the predicted annotations of protein-coding mt genes from *B. graminis* f. sp. *tritici*, *E. pisi,* and *G. cichoracearum*, public RNAseq datasets from these organisms were obtained from NCBI (accessions SRR6026494, SRR7066906 and SRR6232712, respectively). RNAseq reads were mapped to the respective mt genome with HISAT2 v2.1.0 [40] with default settings. Alignments were visualized with IGV v2.5.3 [41] and gene annotations were inspected and adjusted manually.

Genes encoding mt-tRNAs and their secondary structures were obtained with MITOS2 [42]. Introns were classified into group I or group II with RNAweasel [12]. Intronic ORFs were identified with ORFfinder v0.4.3 [43], using as a minimum ORF length 200 bp and genetic code four. ORFs encoding homing endonucleases (HEs) or reverse transcriptases (RTs) were identified and classified based on their conserved domains identified by querying the encoded peptide sequences against the NCBI conserved domain database (CDD) [44] with an e-value <1e-3. Conserved domains within introns were identified by translating the entire intronic sequences in six frames with the *transeq* script from EMBOSS v6.6.0 [45], utilizing the genetic code four and querying the peptide sequences against the NCBI CDD with an e-value <1e-3. Circular representations of the mt genomes were created with Circos v0.69–8 [46]. Tandem repeats were identified with Tandem Repeat Finder v4.09 [47] and overall percentage of repeats of the mt genomes was calculated based on self BLASTn searches, utilizing the parameter *-task blastn* and an e-value <1e-10.

### Identification of introns insertion sites and conservation of intronic sequences

To identify the insertion sites of introns in mt genes, mature transcripts of the genes were aligned with the *mafft-linsi* script from MAFFT v7.455 [48] and the alignments were visualized with SnapGene v5.0.7 (GSL Biotech; available at snapgene.com). Intron insertion sites (i.e. last base-pair position of exons) were reported using *E. necator* C-strain as reference [28]. For better interpretation and visualization, intron insertion sites that differed by at most three base pairs were grouped into one single insertion site. Overall conservation of intronic sequences of the four PM pathogens was determined by pairwise nucleotide alignments produced by the *pairwiseAlignment* function from the R package Biostrings v2.57.2 [49] within R v4.0.3 [50]. To minimize negative impact in the percent identity values due to differences in introns size, the alignment type option in *pairwiseAlignment* was set to *local-global*, which performs a local alignment of the pattern (sequence 1) with a global alignment of subject (sequence 2), where size of subject is at most the size of pattern. Pairwise alignments were processed with the *pid* function from Biostrings [49] to calculate the percent sequence identity considering internal gap positions (option *type=PID1*).

### Nucleotide composition and GC-rich regions of the mt genomes

Distribution of GC content, GC skew [(G-C)/(G+C)] and AT skew [(A-T)/(A+T)] of the mt genomes were determined by calculating the nucleotide composition of non-overlapping sliding windows of 200 bp with the command *comp* of the seqtk v1.3-r106 script (https://github.com/lh3/seqtk). Histograms of GC content were generated within the R software package v4.0.3 [50] and the kernel density estimations were obtained with the *density* function within R, utilizing the Gaussian method. Consecutive 200 bp-windows with GC >50% were merged to estimate length of isochore-like GC-rich regions. To investigate if the GC-rich regions (i.e. windows with GC >50%) from the mt genomes of *G. cichoracearum* and *B. graminis* f. sp. *tritici* migrated from the nuclear genome, the GC-rich regions were queried with BLASTn (e-value <1e-5) against the nuclear genome of *G. cichoracearum* (GCA_003611235.1) and *B. graminis* f. sp. *tritici* (GCA_900519115.1).

### Phylogenetic trees

Phylogenetic trees were built based on multiple sequence alignments generated with the *mafft-linsi* script from MAFFT v7.455 [48]. Alignments were processed with trimAl v1.4 [51] to remove all sites containing gaps. Maximum-likelihood phylogenetic trees were inferred with IQTREE v1.6.11 [52] utilizing ModelFinder [53] to automatically select the best substitution model. Support for branches was obtained based on 1000 ultrafast bootstrap replicates [54]. The phylogenetic tree of the four PM pathogens was constructed based on the concatenated amino acid sequences of 14 protein-coding mt genes, i.e. *atp6*, *atp8*, *nad1*, *nad2*, *nad3*, *nad4*, *nad4L*, *nad5*, *nad6*, *cox1*, *cox2*, *cox3*, *cob*, and *rps3*, utilizing the substitution model cpREV+F+R2. The tree was rooted on *Sclerotinia borealis*. The phylogenetic tree of the *cob* gene from Leotiomycetes was constructed based on coding sequences and utilizing the substitution model TIM+F+G4. The tree was rooted on *Neurospora crassa*. The phylogenetic trees of *cob* genes and ORFs encoding RTs within *cob* introns were constructed based on amino acid sequences and utilizing the substitution models mtZOA+G4 and WAG+F+I+G4, respectively. Trees were visualized and edited with FigTree v1.4.2 (http://tree.bio.ed.ac.uk/software/figtree/).

### Public data acquisition

For comparative analyses, fungal mt genomes were obtained from NCBI Nucleotide and Organelle Genome Resources databases [55] as of 6 May 2021. Nucleotide and protein sequences were obtained from NCBI database utilizing the *efetch* command from NCBI Entrez Direct E-utilities v11.0 [56]. Reads were obtained from NCBI Sequence Read Archive by generating downloadable links with SRA-Explorer online tool (http://sra-explorer.info) based on the accession numbers. Fungal *cob* genes were retrieved from the annotated mt genomes and utilized to mine for additional fungal *cob* genes by querying them against the NCBI nr database using BLASTp with e-value <1e-5 and results restricted to Fungi (taxid 4751).

## Results

### The mt genomes of PMs vary in size but have a similar complement of core mt genes and mt-tRNAs

**Fig. 1.**
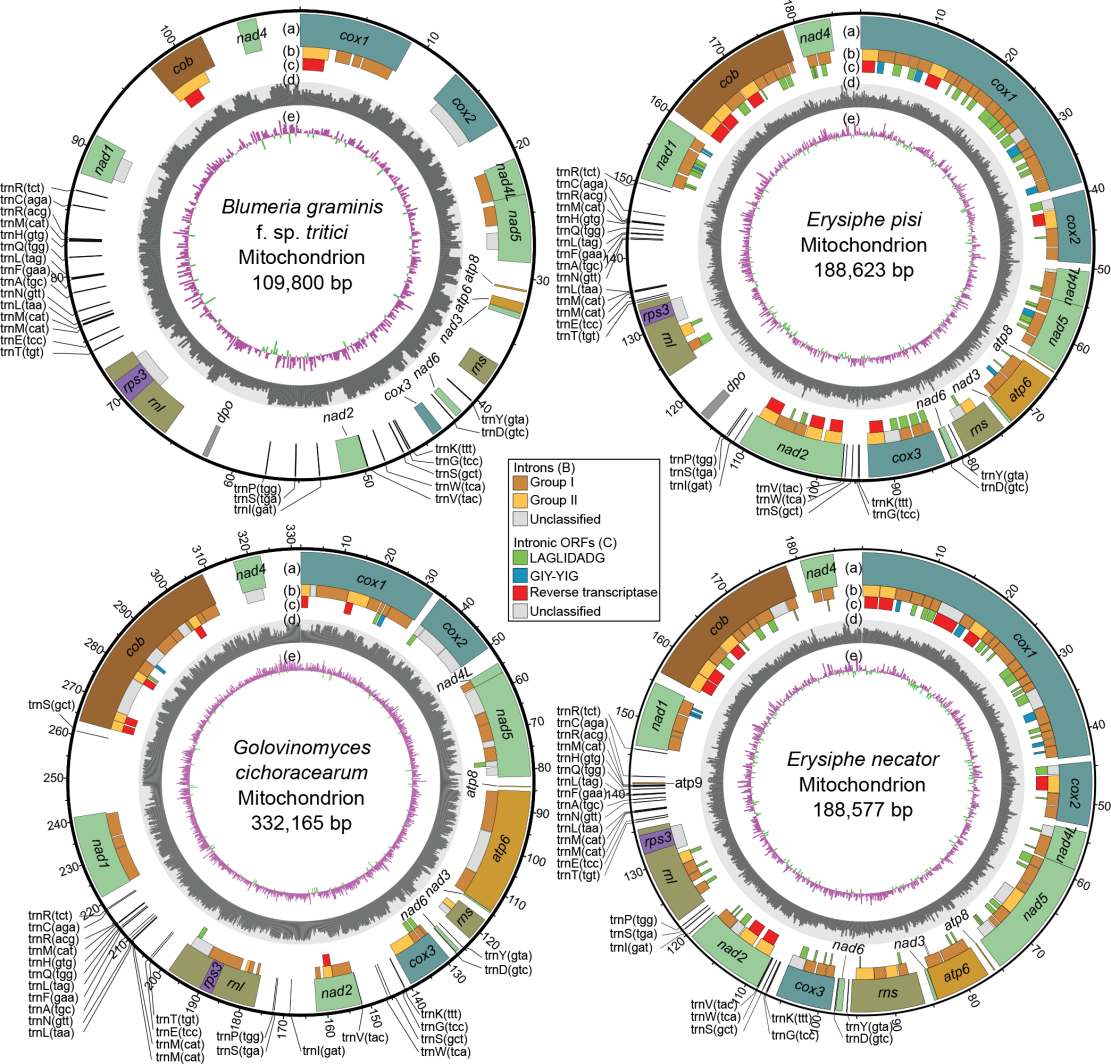
Organization of the mitochondrial (mt) genomes of the powdery mildew fungi *Blumeria graminis* f. sp. *tritici*, *Erysiphe necator*, *Erysiphe pisi*, and *Golovinomyces cichoracearum*. Tracks: (**a**) Core protein-coding and other conserved genes present in the mt genomes of the four powdery mildew species. These include genes encoding the subunits of complex I (*nad1*, *nad2*, *nad3*, *nad4*, *nad4L*, *nad5,* and *nad6*), complex III (*cob*), complex IV (*cox1*, *cox2,* and *cox3*), the ATP-synthase complex (*atp6*, *atp8,* and *atp9*), the small and large ribosomal subunits (*rns* and *rnl*), the ribosomal protein S3 (*rps3*), and a set of mt-tRNAs. Open reading frames (ORFs) encoding DNA polymerase type-B are indicated as *dpo*. (**b**) Introns present in the mt genes of the four powdery mildew species. The introns are classified as group I and group II introns, or as unclassified based on their secondary RNA structures and splicing mechanism. (**c**) ORFs present within introns, encoding homing endonucleases of the LAGLIDADG and GIY-YIG families, or reverse transcriptases. (**d**) GC content scaled from 0–70%. (**e**) GC skew calculated as (**G-**C)/(G+C), with positive and negative values in purple and green, respectively. Both GC content and GC skew were calculated using a non-overlapping sliding window of 200 bp. Data for the mt genome of *E. necator* were obtained from Zaccaron *et al.* (2021) [28] and the circular representation of its mt genome is accordingly adjusted.

To assemble the mt genomes of *B. graminis* f. sp. *tritici*, *E. pisi*, and *G. cichoracearum*, whole-genome sequencing reads of the three species were obtained from NCBI and the reads corresponding to their mt genomes were extracted and assembled (Fig. S1). The resulting mt genomes of *B. graminis* f. sp. *tritici*, *E. pisi*, and *G. cichoracearum* corresponded to single circular DNA sequences of 109800, 188623, and 332165 bp, respectively (Fig. 1). Notably, *G. cichoracearum*, *E. pisi*, and *E. necator* along with the non-PM species *Sclerotinia borealis* (mt genome size of 203051 bp) have the largest mt genomes to date among Leotiomyecetes (Table S1), whereas *G. cichoracearum* further has the second largest fungal mt genome currently available at the NCBI database (Fig. S2).

**Fig. 2.**
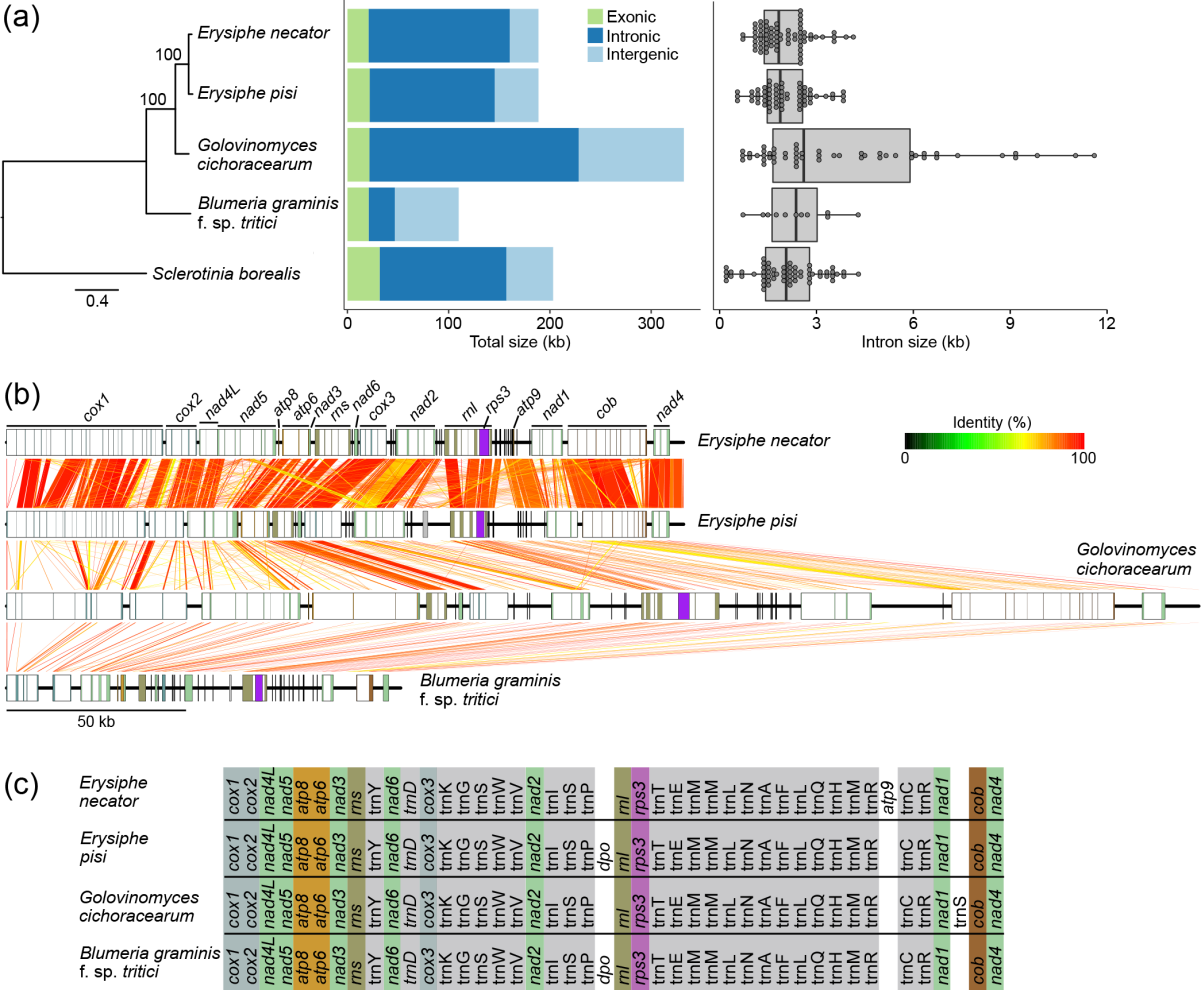
Comparison of the mitochondrial (mt) genomes of the powdery mildew (PM) fungi *Blumeria graminis* f. sp. *tritici*, *Erysiphe necator, Erysiphe pisi,* and *Golovinomyces cichoraceaum*. (**a**) Maximum-likelihood phylogenetic tree constructed based on the amino acid sequences encoded by 14 core mt genes. The tree was rooted on *Sclerotinia borealis,* which is also a species of Leotiomycetes. The bar plot shows the total size of the mt genomes of the four PM species. Bars show the total size of exonic (excluding ORFs encoding homing endonucleases, reverse transcriptases, or DNA polymerases), intronic, and intergenic regions. Differences in mt genome size are due to differences in the size of the intronic and intergenic regions. The box plot depicts the number and length of the introns present in the mt genes of the four PM species. Individual introns are represented by points and are grouped into bins of 150 bp in width. (**b**) The mt genomes of the four PM species are syntenic. Genes are represented as rectangles with intronic regions in white and exonic regions colour-coded as in Fig. 1. Ribbons connect homologous regions identified with BLASTn (e-value <1e-10) and are colour-coded based on the percent of nucleotide identity. (**c**) Mitochondrial gene order in the four PM species is highly conserved. The *rps3* gene is encoded within an intron of *rnl*. The *atp9* gene was predicted in *E. necator* but not in the other three PM species. ORFs encoding putative DNA polymerases type-B are represented as *dpo*. Data for the mt genome of *E. necator* were obtained from Zaccaron *et al.* (2021) [28].

A total of 44, 111, and 58 genes and other ORFs were predicted in the mt genomes of *B. graminis* f. sp. *tritici*, *E. pisi*, and *G. cichoracearum*, respectively, with all genes and ORFs transcribed from the sense strand (Fig. 1). Despite the contrasting number of gene content, all three PM fungi contained single copies of the 13 core mt protein-coding genes involved in the ETC and oxidative phosphorylation, (i.e. *atp6*, *atp8*, *nad1*, *nad2*, *nad3*, *nad4*, *nad4L*, *nad5*, *nad6*, *cob*, *cox1*, *cox2*, and *cox3*), as well as of the two mt rRNA genes (*rns* and *rnl*), and the putative ribosomal protein S3 (*rps3*) gene, located within an intron of *rnl*. The remaining genes corresponded to intronic ORFs predicted to encode homing endonucleases (HEs) of the LAGLIDADG or GIY-YIG families, or reverse transcriptases (RTs), and free-standing ORFs encoding type-B DNA polymerases, present only in *B. graminis* f. sp. *tritici* and *E. pisi* (Table 1). The total size of the exonic sequences of all mt genes, excluding intronic and free-standing ORFs, was also comparable among the three PM species and accounted for 22.1 kb (20.1%) of the mt genome in *B. graminis* f. sp. *tritici*, 22.6 kb (12.0%) in *E. pisi*, and 24.5 kb (7.4%) in *G. cichoracearum*. In *E. necator*, the total size of exonic sequences of all mt genes, excluding intronic and free-standing ORFs, was previously determined to account for 23.3 kb (12.3%) of its mt genome [28], thus agreeing with the other three PM species (Fig. 2a). Finally, also conserved among the three PM species and *E. necator* was the arrangement and orientation of the 13 core mt protein-coding genes in their mt genomes (Fig. 2b, c) . This included *nad4L* and *nad5*, which overlapped by one base in that the last base pair of the stop codon of *nad4L* was the first base pair of the start codon of *nad5,* as well as *atp6* and *nad3*, which were positioned adjacent to each other, an atypical arrangement among fungal mt genomes [28], and were separated by very short intergenic spacers of 46 bp in *B. graminis* f. sp. *titici,* 44 bp in *E. necator* and *E. pisi,* and 40 bp in *G. cichoracearum*.

**Table 1.** Mitochondrial genome statistics of the powdery mildew fungi *Blumeria graminis* f. sp. *tritici* (*Bgt*), *Erysiphe necator* (*En*), *Erysiphe pisi* (*Ep*), and *Golovinomyces cichoracearum* (*Gc*)

Genome features	*Bgt*	En*	*Ep*	*Gc*
Total size (bp)	109800	188577	188623	332165
Overall GC (%)	48.3	33.9	34	45.1
GC-skew (G-C)/(G+C)	0.173	0.101	0.117	0.16
AT-skew (A-T)/(A+T)	0.015	0.031	0.032	0.071
Repetitive DNA (%)	13.6	8	12.7	11.3
Genes	44	107	111	58
mt-tRNAs	25	25	26	25
Introns	11	70	61	53
Group I introns	5	48	42	28
Group II introns	2	13	12	10
Unclassified introns	4	9	7	15
Average intron size (bp)	2359	1992	2025	3900
Intergenic regions size (bp)	62989	28343	43247	103902
Intronic regions size (bp)	25956	139477	123556	206694
Core exonic sequences GC (%)	32.6	32	31.8	33.6
Intergenic regions GC (%)	54.5	38.9	35.9	47.6
Intronic regions GC (%)	45.5	33.2	33.7	45.1
Intronic ORFs	2	65	69	16
LAGLIDADG ORFs	0	44	50	7
GIY-YIG ORFS	0	9	9	2
Reverse transcriptase ORFs	2	11	10	7
Accession	MT880591	MT880588	MT880589	MT880590

*Data for the mitochondrial genome of *E. necator* were obtained from Zaccaron *et al.* (2021) [28].

Despite the overall conservation of the core gene complement among the mt genomes of the four PM species, some differences were noted as well. Specifically, while a transcriptionally active vestigial *atp9* gene that encodes a truncated protein was previously found in *E. necator* [28], no ORF encoding a putative atp9 protein was identified in the other three PM species. Indeed, by querying the *E. necator atp9* coding sequence with BLASTn, homologous sequences were identified in the mt genomes of *E. pisi* and *G. cichoracearum*, but not in *B. graminis* f. sp. *tritici*. However, when translated, the homologous to *atp9* sequence in *E. pisi* contained a premature stop codon, whereas the one in *G. cichoracearum* contained insertions and deletions that caused frameshifts (Fig. S3). Another difference among the four PM species is the presence of an ORF upstream of the *rnl* gene in the mt genomes of *E. pisi* and *B. graminis* f. sp. *tritici* that encodes a putative DNA polymerase type-B (*dpo*), which is absent in *G. cichoracearum* as well as in *E. necator* [28]. Finally, whereas in *B. graminis* f. sp. *tritici*, *E. necator*, and *E. pisi rps3* is located within the last intron of *rnl*, in *G. cichoracearum rps3* is present within the penultimate intron of *rnl*.

In addition to the core mt protein-coding genes, the mt genomes of *B. graminis* f. sp. *tritici*, *E. pisi,* and *G. cichoracearum* contained 25, 25, and 26 mt-tRNA encoding genes, respectively, capable of recognizing the standard set of 20 amino acids. These numbers are once again comparable to the 25 mt-tRNA genes previously found in *E. necator* [28]. As for the core mt genes, the order and orientation of the mt-tRNAs was conserved among the four mt genomes (Fig. 2c), whereas the predicted secondary structures of the identified mt-tRNAs revealed that they all fold into common cloverleaf-like structures (Fig. S4). Most mt-tRNA genes identified in the mt genomes of *B. graminis* f. sp. *tritici*, *E. pisi,* and *G. cichoracearum* were single-copy. Exceptions were the mt-tRNAs that decode arginine (*trnR*), leucine (*trnL*), methionine (*trnM*), and serine (*trnS*) that were present in two, two, three, and two copies, respectively, in the mt genomes of *B. graminis* f. sp. *tritici* and *E. pisi*, whereas *G. cichoracearum* also contained the same number of copies of *trnR*, *trnL,* and *trnM* but had a third copy of *trnS* between *nad1* and *cob*.

### Repetitive DNA content and mt introns are poorly conserved among PMs

Although coding sequences of the core mt genes were comparable in size among the three PM species analysed in this study and *E. necator* [28], large differences were found in their repetitive DNA content as well as their intronic and intergenic regions (Fig. 2a, Table 1). Specifically, repetitive DNA accounted for 13.6, 12.7, and 11.3% of the mt genomes of *B. graminis* f. sp. *tritici*, *E. pisi*, and *G. cichoracearum,* which also harboured 142, 97, and 206 short tandem repeats, respectively (Table S2). This contrasts with the mt genome of *E. necator*, which has a repetitive DNA content of 8% and 45 short tandem repeats [28], and suggests that repetitive DNA contributes to a greater extend to the organization of the mt genomes of *B. graminis* f. sp. *tritici*, *E. pisi*, and *G. cichoracearum,* as compared to *E. necator*.

**Fig. 3.**
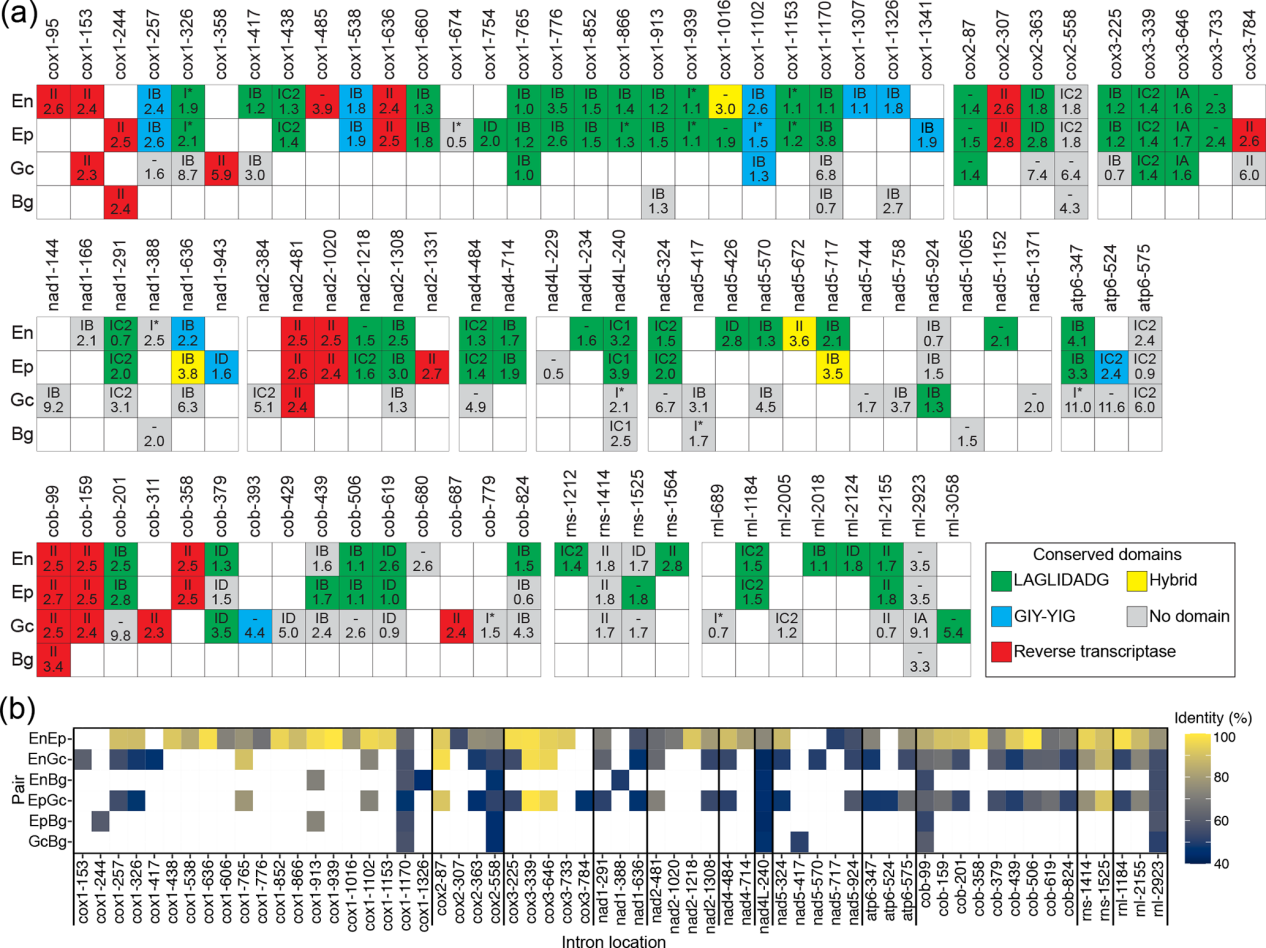
Intron insertion sites in mitochondrial (mt) genes of the powdery mildew (PM) fungi *Blumeria graminis* f. sp. *tritici, Erysiphe necator, Erysiphe pisi,* and *Golovinomyces cichoracearum*. (**a**) Introns are depicted as colour-coded square boxes and their insertion sites within respective genes are shown as columns. Base-pair coordinates are given at the top and are in reference to the coding sequences of the *E. necator* mt genes. Intron type and length (kb) are shown inside the boxes, at the top and bottom, respectively. Colours indicate the absence (grey boxes) or presence (coloured boxes) of conserved domains in the introns. Conserved domains include homing endonucleases (HEs) from the LAGLIDADG (green) or GIY-YIG families (blue boxes), reverse transcriptases (RTs) (red boxes), and hybrid LAGLIDADG/GIY-YIG or GIY-YIG/RT domains (yellow boxes). The introns inserted at cox1-1016, *nad1*-636, and nad5-717 positions contain a hybrid LAGLIDADG/GIY-YIG domain, and the intron inserted at the nad5-672 position contains a hybrid GIY-YIG/RT domain. Absence of introns is indicated with blank white boxes. Genes without introns in all four species are not shown. The figure shows that intron content and intron insertion sites within orthologous mt genes vary widely among the four PM species, but are correlated to the phylogenetic relatedness of the species. (**b**) Heat-map showing the pairwise nucleotide identity between introns inserted in the same site within orthologous mt genes of the four PM species. En: *E. necator*; Ep: *E. pisi*; Gc: *G. cichoracearum*; Bg: *B. graminis* f. sp. *tritici*. As for other intron features, introns inserted in the same site share limited primary sequence identity that is largely defined by the phylogenetic relatedness of the species. The data for the mt genome of *E. necator* were obtained from Zaccaron *et al.* (2021) [28].

Large differences in intron content were observed as well among the mt genomes of the four PM species, with variations extending to the number, density, size, and distribution of introns hosted within orthologous mt genes as well as conserved domains present in these introns (Fig. 3a). Specifically, a total of 11, 61, and 53 introns were identified in the mt genomes of *B. graminis* f. sp. *tritici, E. pisi,* and *G. cichoracearum* (Table S3). Intron density was also lowest for *B. graminis* f. sp. *tritici* (0.7 introns per kb of cds)*,* followed by *G. cichoracearum* (3.3 introns per kb of cds), and *E. pisi* (4.0 introns per kb of cds). The number of introns previously identified in the mt genome of *E. necator* was 70, with an intron density of 4.4 introns per kb of cds [28]. This indicates that intron features vary more homogeneously between *E. necator* and *E. pisi* rather than between the other species-pairs (Fig. 3a). A weak correlation (*R*=0.49, *P*-value=0.51) existed between intron numbers and mt genome size among the four PM species (Fig. S5a), whereas a stronger correlation (*R*=0.95, *P*-value=0.055) was observed between the total size of introns and mt genome size (Fig. S5b), indicating that intron size, rather than intron numbers, dictated the variation in mt genome size among the four PM species. This conclusion is also supported by the differences in size among orthologous genes in the four PM species (Table S4), which are largely explained by the size of their introns (*R*=1, *P*-value<2.2e-16) (Fig. S5c).

Next to their differences in size, introns inserted at the same sites within orthologous genes often shared limited primary sequence identity as well, indicating poor intron conservation among the four Erysiphales (Fig. 3b, Table S5). The most conserved intron among the four PM species was the intron inserted at position 99 of the cytochrome *b* gene (*cob*) cds (cob-99), with an average pairwise identity of 63.0%, indicating that there are no introns that are highly conserved among all four species. The poor conservation of introns inserted at the same sites within orthologous genes further extended to the presence/absence of ORFs embedded in them. Indeed, ORFs encoding HEs or RTs were found in 69 (85.2%) and 65 (82.8%) of the introns in *E. pisi* and *E. necator*, respectively, but only in 16 (30.2%) and two (18.2%) of the introns in *G. cichoracearum* and *B. graminis* f. sp. *tritici,* respectively (Fig. 3a). These results indicate that *E. pisi* (1.13 ORFs/intron) and *E. necator* (0.93 ORFs/intron) were richer in intronic ORFs as compared to *B. graminis* f. sp. *tritici* (0.18 ORFs/intron) and *G. cichoracearum* (0.30 ORFs/intron). Overall, of the 61 introns (42 group I, 12 group II, and seven unclassified) detected in the mt genome of *E. pisi*, 42 contained HEs of the LAGLIDADG (*n*=36) or GIY-YIG (*n*=8) families, and ten contained RT-encoding ORFs. In an analogous way, *B. graminis* f. sp. *tritici* contained two group II introns containing RT-encoding ORFs, but none HE-encoding ORF, whereas *G. cichoracearum* had seven RT-encoding ORFs within its group II introns and five group I introns with HE-encoding ORFs of the LAGLIDADG (*n*=4) or GIY-YIG (*n*=1) families (Fig. 3a).

### The mt genomes of *B. graminis* f. sp. *tritici* and *G. cichoracearum* show a bimodal distribution of GC content.

High variation in GC content was observed among the mt genomes of the three PM species, ranging from 34.0% in *E. pisi*, 45.1% in *G. cichoracearum*, and 48.3% in *B. graminis* f. sp. *tritici*, which indicates substantial differences in nucleotide composition (Table 1). Assessment of GC content separately in gene coding and non-coding sequences indicated that exonic sequences in *B. graminis* f. sp. *tritici, E. pisi,* and *G. cichoracearum* exhibited an average GC content of 32.6, 31.8, and 33.6%, respectively, whereas intergenic and intronic sequences exhibited an average GC content of 51.9, 34.3, and 45.9%, respectively (Table 1). The overall GC content of the *E. necator* mt genome was previously determined as 33.8%, with exonic and intronic sequences exhibiting an average GC content of 32.0 and 34.1%, respectively [28]. Collectively, these results indicate that the differences in GC content among the four Erysiphales are mainly driven by nucleotide compositional differences between functional and non-functional sequences.

**Fig. 4.**
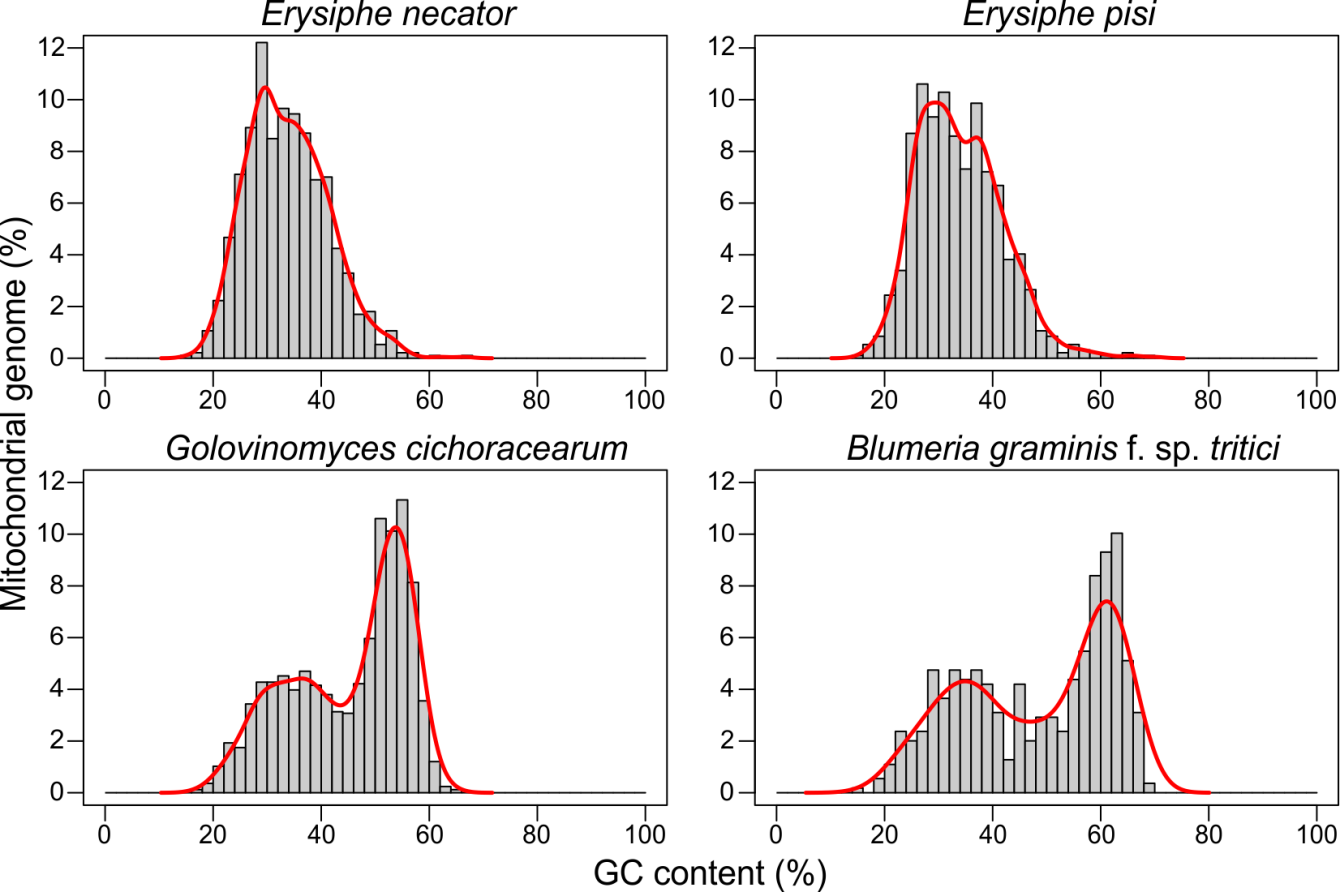
GC content comparison among the mitochondrial (mt) genomes of the powdery mildew fungi *Blumeria graminis* f. sp. *tritici*, *Erysiphe necator, Erysiphe pisi,* and *Golovinomyces cichoracearum*. Histograms depict the percentage of the respective mt genome (Y-axis) containing the indicated GC content (X-axis). Solid lines represent estimated distributions based on the kernel density of the histograms. The graphs show that the mt genomes of *E. necator* and *E. pisi* have a unimodal distribution of GC content with a peak at approximately 29%, whereas the mt genomes of *G. cichoracearum* and *B. graminis* f. sp. *tritici* exhibit a bimodal distribution of GC content, with two peaks at 36 and 53%, and 35 and 61%, respectively. Data for the mt genome of *E. necator* were obtained from Zaccaron *et al.* (2021) [28].

An inspection using a sliding window analysis of the variation in the GC content across the four mt genomes further showed that GC content was unimodally distributed in the mt genomes of *E. necator* and *E. pisi*. In contrast, the mt genomes of *B. graminis* f. sp. *tritici* and *G. cichoracearum* exhibited a bimodal distribution, characterized by interspersed isochore-like regions of GC-poor (i.e. GC of 20–40%) and GC-rich (i.e. GC of 50–65%) content (Fig. 4). The GC-rich regions were longer in *B. graminis* f. sp. *tritici* (average of 837 bp) and covered a higher fraction of its mt genome (52.6%) compared to *G. cichoracearum* (average length of 450 bp, covering 45.9% of its mt genome). In contrast, only 2.9 and 3.7% of the mt genomes of *E. necator* and *E. pisi*, respectively, had GC content higher than 50%. As expected, almost all of the GC-rich regions in the mt genomes of *B. graminis* f. sp. *tritici* (66 out of 69 regions) and *G. cichoracearum* (230 out of 236 regions) were located within intergenic regions or introns and did not appear to affect neighbouring coding sequences. Repetitive DNA identification performed with RepeatMasker showed that none of the GC-rich regions contained interspersed repeats, whereas simple repeats and low complexity regions accounted for only 7.5% of the GC-rich regions in *B. graminis* f. sp. *tritici* and 3.3% of the regions in *G. cichoracearum*, suggesting that the GC-rich regions are not composed of repetitive DNA. Finally, homology searches performed with BLASTn (e-value <1e-5) further revealed that none of the GC-rich regions was conserved between *G. cichoracearum* and *B. graminis* f. sp. *tritici*, whereas no evidence of a possible migration of these sequences from their respective nuclear genome were obtained as well.

To further inquire on the origin and evolution of the GC-rich regions in *B. graminis* f. sp. *tritici* and *G. cichoracearum*, those that did not overlap with functional sequences were then queried in BLASTn searches against the NCBI nr database. From the 66 GC-rich regions present in *B. graminis* f. sp. *tritici* queried, 58 had only a single hit on one of the scaffolds of the *B. graminis* f. sp. *tritici* assembly containing its mt genome. The other eight regions had somewhat significant hits (e-value <1e-30) to sequences from a wide range of species, including fungi and animals (Table S6). When querying the 230 GC-rich regions present in *G. cichoracearum*, then 212 had hits solely to scaffolds from a genome assembly of the oomycete *Albugo laibachii*. However, further blast searches revealed that this same *Albugo laibachii* genome assembly contained a contig (FR825110.1) highly homologous (99% nucleotide identity) to the internal transcribed spacer (ITS) sequences from *Golovinomyces* spp. Taken together, these data suggest that the GC-rich isochore-like regions present in the genomes of *B. graminis* f. sp. *tritici* and *G. cichoracearum* are likely to have been formed *de novo* in each of the two species or to have been transmitted vertically rather than horizontally.

Bimodal distribution of GC content has been previously described in nuclear genomes of fungi, but it is hardly ever reported in fungal mt genomes. Therefore, we next examined the patterns of GC content variation in 23 mt genomes from phylogenetically related species of Leotiomycetes. However, only the genome of *Blumeria graminis* f. sp. *hordei* was organized into isochore-like regions of high-GC and low-GC regions, as were the mt genomes of *B. graminis* f. sp. *tritici* and *G. cichoracearum* (Fig. S6). We then extended our analysis to 949 mt genomes from phylogenetically diverse fungi, with the stipulation that they should have an average GC content of 40% or higher. Surprisingly, from the 949 fungal mt genomes analysed, only 23 from 21 different species, representing four phyla, had GC ≥40%, indicating that fungal mt genomes are predominately AT-rich (Fig. S7, Table S1). However, the majority (*n*=17) of these 23 mt genomes had a single GC peak between 40–60%, indicative of a homogeneous GC content and a unimodal distribution (Fig. S8). The mt genomes of only seven species (i.e. *B. graminis* f. sp. *hordei, Magnusiomyces tetraspermus, Morchella crassipes, Alternaria alternata, Pyrenema ompalodes, Synchytrium endobioticum,* and *Glomus cerebriforme*) exhibited GC content distribution patterns suggestive of bimodal organization but only in *B. graminis* f. sp. *hordei* the heterogeneity in GC content was as pronounced as in *B. graminis* f. sp. *tritici* and *G. cichoracearum* (Fig. S8). Taken together, these data indicate that *B. graminis* f. sp. *tritici* and *G. cichoracearum* display a rather idiosyncratic mode of bimodality in GC content in their mt genomes.

### Erysiphales have large cytochrome *b* genes with rare introns embedded in them that contain RT-encoding ORFs

**Fig. 5.**
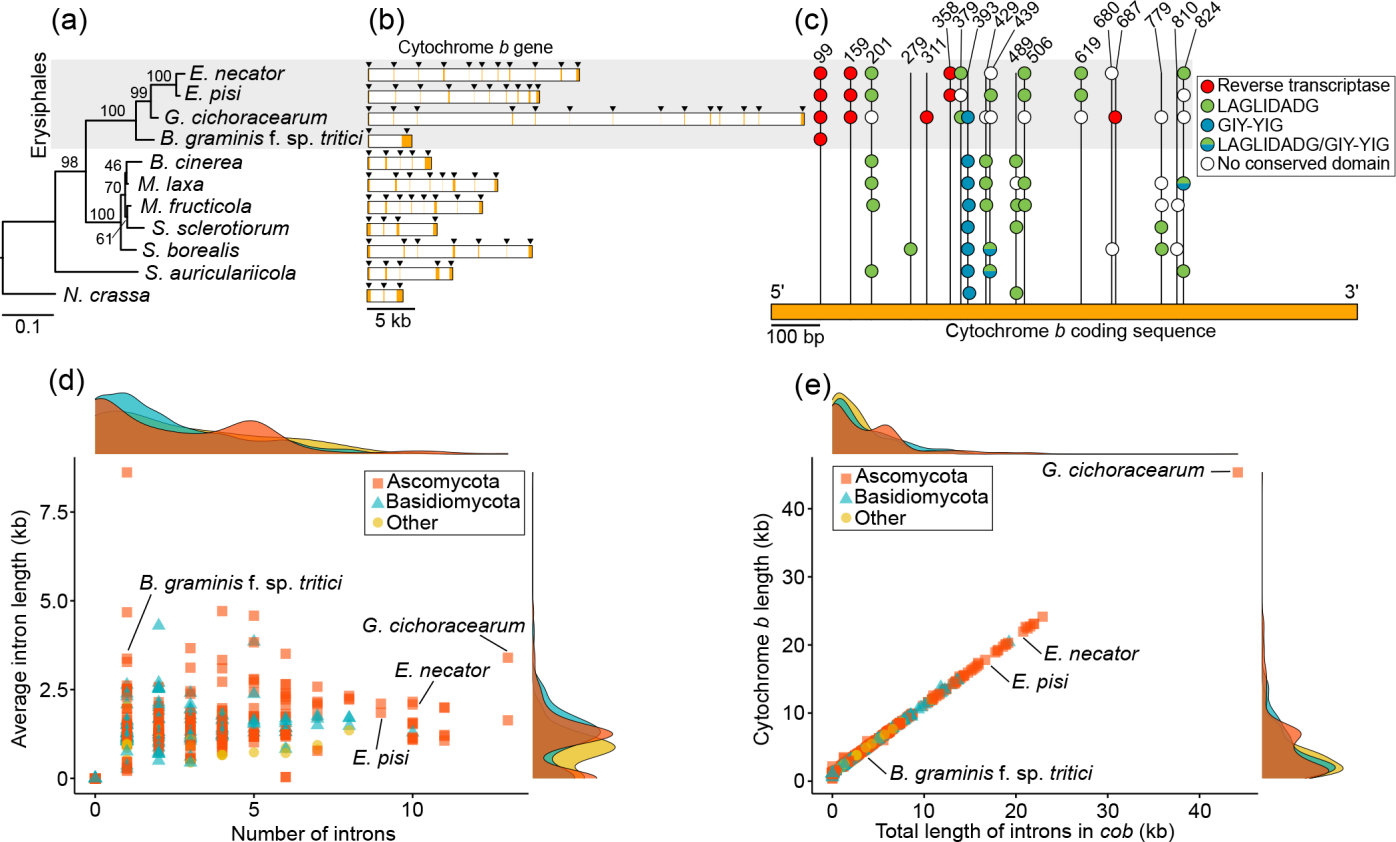
Comparative analysis of cytochrome *b* (*cob*) genes from the powdery mildew fungi *Blumeria graminis* f. sp. *tritici*, *Erysiphe necator, Erysiphe pisi,* and *Golovinomyces cichoracearum,* and other fungal species. (**a**) Maximum-likelihood phylogenetic tree constructed based on *cob* coding sequences from ten species of Leotiomycetes and the outgroup species *Neurospora crassa*. (**b**) *Cob* gene structure from the eleven species used to construct the phylogenetic tree. Exons and introns are represented by orange and white bars, respectively. For clearer visualization, exons are also indicated with black triangles. The image shows that *cob* genes differ dramatically in size and intron content among species of Leotiomycetes. (**c**) Insertion sites of the introns present in the *cob* genes of the eleven species used in our analysis and the conserved domains encoded within these introns. Insertion sites are represented by vertical lines and presence of introns is indicated by circles colour-coded based on their conserved domain content. This figure shows that species of Erysiphales are the only ones among the Leotiomycetes that contain introns with reverse transcriptases (RTs) embedded in them, and that intron insertion sites are poorly conserved between them and the rest of the Leotiomycetes. (**d, e**) Scatterplots that show the average intron length in *cob*, number of introns in *cob*, total length of *cob*, and total length of introns in *cob*, among 703 *cob* genes of fungal species of Ascomycota, Basidiomycota, and ‘other’ fungal phyla (i.e. Blastocladiomycota, Chytridiomycota, Cryptomycota, Mucoromycota, and Zoopagomycota) included in the analysis. The scatterplots shows that with the exception of *B. graminis* f. sp. *tritici,* Erysiphales have unusually large and intron-rich *cob* genes. Data for the mt genome of *E. necator* were obtained from Zaccaron *et al.* (2021) [28].

The cytochrome *b* (*cob*) gene receives particular attention in phytopathogenic fungi as point mutations in this gene are associated with resistance to QoI fungicides [18]. The *cob* genes of *B. graminis* f. sp. *tritici, E. necator*, *E. pisi,* and *G. cichoracearum* exhibited fairly conserved coding sequences, both in size (1161 bp, 1170 bp, 1170 bp, and 1170 bp, respectively) and nucleotide identity (89.4–97.7%) (Table S7). In contrast, a large variation was seen in the size (4.5 to 45.3 kb) and number of introns (1 to 13) present in them [28] (Fig. 5a, b, Table S7), whereas pairwise alignments of homologous introns further showed that they exhibit a much broader range of nucleotide identities as compared to the *cob* coding sequences (Fig. 3b). However, despite such differences in size and primary sequence identity, some notable similarities in the distribution and content of introns present in the *cob* genes of *E. necator*, *E. pisi,* and *G. cichoracearum* were observed as well. The *cob* gene in *B. graminis* f. sp. *tritici* stood apart from its orthologs in the other three PM species, as it contained only one intron (cob-99), which was conserved among all four PM species (Fig. 5c). Intron insertion sites in the *cob* genes of *E. necator*, *E. pisi,* and *G. cichoracearum* were fairly conserved, with nine sites shared between *E. necator* and *E. pisi*, and eight sites being conserved in all three species (Fig. 5c). Notably, some of the differences in intron insertions sites were found around codons 129, 137, and 143, which affect amino acids that when mutated confer resistance to QoI fungicides [18]. Specifically, the intron cob-429 that is inserted at codon 143 and its presence blocks the formation of the p.G143A substitution [57], was present in *G. cichoracearum* but not in the other three PM species. One more intron, cob-393, was found six base pairs downstream of codon 129 and was present only in *G. cichoracearum,* whereas a third intron, cob-379, was inserted five base pairs upstream of codon 129 and was present in *E. necator*, *E. pisi,* and *G. cichoracearum* (Fig. 5c). Although these introns do not directly affect codon 129, it is plausible that their mis-splicing may indirectly affect this codon, and thus QoI resistance as well.

In order to further identify structural differences among the *cob* genes of the PM species examined in this study and of other phylogenetically closely related fungal species, we obtained this gene from 19 other members of Leotiomycetes outside the Erysiphales (Table S8). The size of the *cob* gene varied greatly among these 19 species, ranging from 1.1 kb in *Rhynchosporium orthosporum* to 17.1 kb in *Sclerotinia borealis*. Thirteen species had no introns in their *cob* genes, whereas the remaining six species had three to six introns (Fig. 5a, b). Interestingly, intron insertion positions in *cob* were somewhat conserved within Leotiomycetes, as the 61 introns identified were inserted into 18 distinct sites between positions 99 and 824 of the *cob* cds (Fig. 5c). Of these, insertion sites 99, 159, 311, 358, 379, 619, and 687 were unique to the Erysiphales, whereas from the remaining 11 insertion sites, eight were shared between at least one species of Erysiphales and one other species of Leotiomycetes. Among the shared intron insertion sites was cob-429, which was present in *G. cichoracearum*, *B. cinerea, Monilinia laxa*, and *M. fructicola*. Notably, group II introns with RT domains were present in five different sites within *cob* and were exclusive to the Erysiphales. Moreover, the intron cob-393 containing a GIY-YIG HE domain that was present in *G. cichoracearum* but absent in *B. graminis* f. sp. *tritici*, *E. necator*, and *E. pisi*, was conserved in all other analysed members of the Leotiomycetes and in *Neurospora crassa* (Fig. 5c). Taken, together, these results indicate that the *cob* genes of PM species are rather unique with respect to their intron content among Leotiomycetes.

### Comparative analysis of fungal cytochrome *b* genes further highlights their idiosyncratic nature in *E. necator*, *E. pisi*, and *G. cichoracearum.*

To probe further into the idiosyncrasy of the *cob* genes from the three Erysiphales, we then extracted this gene from fungal mt genomes available at NCBI. A total of 703 *cob* genes were retrieved, representing 254 fungal genera from seven phyla (Table S8). Overall, intron abundance among *cob* genes was low, with 526 (74.8%) of the genes having four or less introns, and 218 (31.0%) having no introns (Fig. 5d). Average intron density (i.e. the number of introns per kb of cds) among the 485 *cob* genes with at least one intron was 3.1, which is considerably lower than the average intron density of *cob* genes from the three PM species (9.1) or from all four together (7.1). Overall, *G. cichoracearum* and the yeast *Metschnikowia amazonensis* possessed the highest number of introns (*n*=13) in *cob* among all 703 fungal mt genomes analysed (Fig. 5d). Seventeen other fungal mt genomes had a total of nine or more introns in their *cob* gene. Among them, there were 13 yeast species of the *Metschnikowia*/*Candida* genera (9–11 introns), *E. necator* (ten introns), *Agaricus bisporus* (ten introns), *Juglanconis juglandina* (ten introns), and *E. pisi* (nine introns). Next to having the largest number of introns in *cob*, *G. cichoracearum* also had the largest *cob* gene (45.3 kb) among the 703 fungal *cob* genes analysed, followed by *Morchella importuna* (24.1 kb) (Fig. 5e). These results indicate that, compared with other fungal species, *G. cichoracearum*, *E. necator*, and *E. pisi* have unusually large *cob* genes that are rich in introns.

**Fig. 6.**
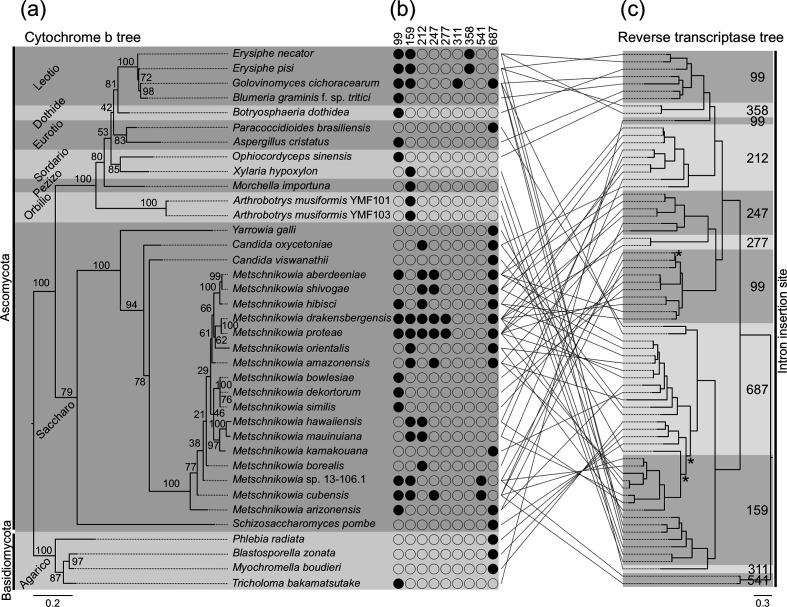
The cytochrome *b* (*cob*) genes from the powdery mildew fungi *Blumeria graminis* f. sp. *tritici*, *Erysiphe necator, Erysiphe pisi,* and *Golovinomyces cichoracearum* contain rare intronic ORFs encoding reverse transcriptases (RTs). (**a**) Maximum-likelihood phylogenetic tree obtained based on the translated amino acid sequences of all fungal *cob* genes containing RT-encoding ORFs among 703 fungal mitochondrial genomes analysed. The taxonomic classes of the fungal species are shown on the left. (**b**) Insertion sites in their respective *cob* cds of introns harbouring RT-encoding ORFs. Presence and absence of RT-encoding ORFs are indicated with filled and blank circles, respectively. (**c**) Maximum-likelihood phylogenetic tree obtained based on the translated amino acid sequences of RT-encoding ORFs present within *cob* introns. Insertion sites of the introns harbouring the RT-encoding ORFs are shown on the right. For clearer visualization, support bootstrap values were omitted. Branches with low bootstrap support (<75%) are labelled with an asterisk (*). Pairwise comparison of the two trees shows multiple cases of phylogenetic incongruences that could be indicative of horizontal gene transfer events. All intron insertion sites are reported using *E. necator* as reference. Data for *E. necator* were obtained from Zaccaron *et al.* (2021) [28].

The same dataset of 703 fungal mt genomes was next utilized to search for species that, similar to the four PMs analysed in this study, have introns in their *cob* genes with RT-encoding ORFs (NCBI accession cd01651) embedded in them. Surprisingly, next to the four PMs, only 33 more fungal species had a *cob* gene with at least one intron containing an RT domain (Fig. 6a). The fungal species were from taxonomically diverse classes and typically each taxonomic class will be represented by only one to four species. However, two notable exceptions for which the presence of an RT domain within *cob* introns is common are the PMs and the yeast *Metschnikowia* spp., as all four PM species and ten of the 17 of *Metschnikowia* spp. included in the analysis had such domains. Moreover, while PMs harboured up to four *cob* introns with an RT-encoding ORF (*G. cichoracearum*) and *Metschnikowia* spp. up to six introns, other fungal species typically harboured a single *cob* intron with an RT domain. Collectively, this data indicate that the two lineages differ from other fungi by containing RT-encoding ORFs in multiple introns within *cob*. Finally, a total of 73 RT-encoding ORFs were identified within the introns of the 37 *cob* genes (Table S9). These introns were inserted into nine different sites within the *cob* cds, with insertion sites cob-99 and cob-687 being the most common ones, as they harboured 36 (49.3%) of the 73 RT-encoding ORFs identified (Fig. 6b). Interestingly, a phylogenetic tree constructed using the translated RT-encoding ORFs revealed grouping based on the insertion site of the introns that contained them in the *cob* gene rather than their respective species phylogeny, suggesting that some of these domains could have been acquired through horizontal transfer (Fig. 6c).

## Discussion

In this study, we assembled the mt genomes of three PM species and further compared them with the mt genome of a fourth species, in order to reveal contrasting differences among mt genomes of PM species, and to show idiosyncratic features of their mt genomes as compared to those of other fungal species. Our comparative analysis revealed a markedly variability in mt genome sizes among PM species, which ranged from 109800 bp to 332165 bp. The 109.8 kb mt genome of *B. graminis* f. sp. *tritici* was the smallest among the four PM analysed, and is comparable in size with the mt genomes of *B. graminis* f. sp. *hordei* isolates DH14 (106.3 kb) and RACE1 (139.3 kb) described previously [31]. The seemingly larger genome size of *B. graminis* f. sp. *hordei* RACE1 is caused by a 32.2 kb duplication at the immediate ends of the contig representing the mt genome of this isolate [31]. However, this duplication is likely the result of untrimmed overlapping ends, which often occur during assembly of circular genomes, and thus by removing this duplication then the size of the mt genome of the *B. graminis* f. sp. *hordei* RACE1 isolate is reduced to 107.1 kb, which is comparable to other mt genomes of *B. graminis* [31]. Recently, a 26.0 kb mt genome for the PM *Podosphaera xanthii* has been described [58], which is considerably smaller than the PM mt genomes presented herein. However, a phylogenetic analysis failed to group the *Podosphaera xanthii* mt genome with members of the Leotiomycetes [58], suggesting that this mt genome is not from a PM species. The largest fungal mt genomes reported to date are those of the so-called ‘true morels’ fungi *Morchella importuna, M. conica,* and *M. crassipes,* sized at 272238 bp [59], 280763 bp [60], and 531195 bp [6], respectively. Thus, at 332165 bp, the mt genome of *G. cichoracearum* currently stand as the second largest among fungi and the largest among pathogenic fungi. With the advent of new cost-effective sequencing technologies, this pattern will likely continue and even larger fungal mt genomes will possibly be reported, thus highlighting the variable and dynamic nature of fungal mt genomes.

Our analyses indicated that the variation in size among the mt genomes of the four Erysiphales examined in this study is mainly due to differences in the number and length of their intergenic and intronic regions. In contrast, their core protein-coding mt genes were mostly conserved and showed no evidence of gene duplication. Also, no other genomic rearrangements were observed, although these can even occur among fungal species of the same genus [61]. A recent study, which analysed gene rearrangements in the mt genomes of 16 species of Leotiomycetes, identified five distinct groups, indicating that Leotiomycetes have undergone major gene rearrangements during their evolutionary history [62]. Interestingly, the gene arrangement present in the four Erysiphales analysed in this study is not in agreement with any of those described in the other 16 Leotimycete species [62], and thus constitutes a novel type of mt gene arrangement within Leotiomycetes. A noteworthy discrepancy of the Erysiphales is that the *nad2* and *nad3* genes are not placed next to each other, as is the case in several fungal species [8, 28], including in the 16 species of Leotiomycetes analysed before [62]. This suggests that this unusual rearrangement occurred after divergence of the Erysiphales, and the gene order was maintained during subsequent speciation events.

Next to differences in mt intron size and numbers, intron content was also very different among the four PM species. Homing endonuclease genes (HEGs) are selfish genetic elements that spread at a super-Mendelian rate within a population [13]. They are thought to have no effect on the fitness of the host organism, and therefore are not subject to natural selection. Once fixed in a population, these elements accumulate mutations that eventually disrupt their ability to spread. Their preservation during evolution is ensured by a cyclical model of acquisition, degeneration and loss, in which HEGs constantly move to new species by horizontal transfer before they degenerate, whereas the degenerated HEGs in the donor species are eventually lost from its genome [13, 15]. This loss is mainly through a mechanism of precise excision, mediated by reverse transcription of spliced RNA. As a result, it leads to the re-constitution of the sequence recognized by the HE, thus making the sequence susceptible again to re-invasion, and starting the process of acquisition, degeneration, and loss of HEGs. This cyclical model of HEG propagation could explain the marked intron content variability among Erysiphales, which suggests extensive intron gain and/or loss throughout their evolutionary history. Another explanation for the observed intron variability is the likelihood of a species to acquire horizontally transferred sequences. It is believed in this respect that HEGs are more commonly found in organelles of eukaryotes of simpler organization, such as fungi, algae and protists, because of the ease of horizontal transmission among these taxa, for which access to the germline is more effortless [63]. However, this scenario would imply that the mt genome of *B. graminis* f. sp. *tritici* would be less susceptible to horizontal transmission compared to the mt genomes of *E. necator*, *E. pisi*, and *G. cichoracearum*. Although a definite answer cannot be given, both horizontal transmission of HEGs and their degeneration could be evoked to explain the different conservation levels observed among introns.

Another interesting feature observed in the mt genomes of *B. graminis* f. sp. *tritici* and *G. cichoracearum* was an unusual bimodal GC distribution that was absent in *E. necator*, *E. pisi,* and other Leotiomycetes. The high GC content regions had no apparent impact on coding sequences, because they were almost exclusively located within intronic or intergenic regions. Lack of homology between these high-GC regions of *B. graminis* f. sp. *tritici* and *G. cichoracearum* does not support inheritance from a common ancestor, but may alternatively suggest acquisition by horizontal transfer. Also, homology searches performed with BLASTn against the NCBI nr database indicated that these regions were not well conserved in other species, except in the oomycete *A. laibachii*, which contained sequences highly homologous to the high-GC regions from *G. cichoracearum*. As with PM pathogens, *A. laibachii* is also an obligate biotrophic pathogen that causes downy mildew on *Arabidopsis thaliana*. Previous studies have shown that both *G. cichoracearum* and *G. orontii* can also infect *A. thaliana* [27, 64], and thus sharing the same host could facilitate horizontal transmission between *G. cichoracearum* and *A. laibachii*. However, this hypothetical horizontal transmission might not be the case because in the *A. laibachii* genome assembly we identified a scaffold that matches the ITS sequence from *Golovinomyces* spp. This raises the possibility of contamination by *Golovinomyces* spp. of the sequenced sample of *A. laibachii*. Nevertheless, the possible origin of these high-GC regions in *G. cichoracearum* and *B. graminis* f. sp. *tritici* remains unclear and future studies may shed light into their formation and impact on the mt genome.

A recent study identified 21 intron insertion sites within *cob* among 129 fungal species of the Ascomycota [65]. In the present study, among only nine representatives of the Leotiomycetes, we identified 18 insertion sites, seven of which were unique to Erysiphales. Interestingly, the most commonly found insertion sites in fungal *cob* genes were at position 393 and 490 [65]. However, while these insertion sites were common among other Leotiomycetes, they were rare among Erysiphales. Instead, the most common intron within Erysiphales was at position 99, which was absent in other Leotiomycetes and was not reported in the study presented in [65]. Concomitant to rare intron insertion sites, RT-encoding ORFs were also common in Erysiphales. Although, the presence of RT-encoding ORFs in fungal mt introns has been frequently reported, we show that within fungal *cob* introns these ORFs are rare, except among the Erysiphales and *Metschnikowia* spp. In addition, a phylogenetic analysis revealed that the RT-encoding ORFs group based on their respective insertion site, which differs substantially from their respective host species phylogeny. This contrasting phylogenetic placement is indicative of horizontal transfer and is in accordance with the hypothesized cyclical model of HEG acquisition, degeneration, and loss.

The presence of an intron adjacent to codon 143 of the *cob* gene (i.e. intron cob-429) has been shown to prevent the p.G143A mutation in fungal pathogens. Mutation at this codon can interfere with the splicing of the intron, which consequently leads to a presumably deficient *cob*. This mechanism has been previously reported in the Leotiomycetes *B. cinerea* [19] and *M. fructicola* [20]. Particularly, two different *cob* alleles were identified among isolates of *B. cinerea* based on the presence or absence of the cob-429 intron. This same study revealed that individuals carrying the p.G143A mutation did not have the cob-429 intron, supporting the hypothesis that this intron prevents resistance to QoI fungicides acquired by the p.G143A mutation. In accordance with previous studies, the presence of the cob-429 intron was also detected in *G. cichoracearum*, suggesting that isolates of the fungus bearing this intron are unable to acquire resistance to QoIs trough the p.G143A mutation. Further studies can reveal the frequency of this intron among populations of *G. cichoracearum*.

In summary, in this study we produced high-quality mt genomes for three economically important PM pathogens. By doing so, we provided novel insights into the mt genome organization of members of the Erysiphales and expanded the spectrum of mt genomic resources for these pathogens. Notably, the mt genomes of PM pathogens are highly syntenic but vary greatly in size. *Blumeria graminis* f. sp. *tritici* and *G. cichoracearum* also differed substantially from other fungal species by having an unusual bimodal GC content with low GC regions interspersed with high GC regions. In addition, mt genomes of the four PM pathogens differed from other fungi by having atypical RT-encoding ORFs within the *cob* gene. The analysed mt genomes of Erysiphales also presented a dynamic architecture of presence/absence of introns and intronic ORFs encoding HEs and RTs. In this context, future studies can elucidate how active HEs and RTs are and their evolutionary impact in the mt genomes of the analysed Erysiphales.

## Supplementary Data

Supplementary material 1

Supplementary material 2
