# Supplementary material for: Characterization of the mitochondrial genomes of three powdery mildew pathogens reveals remarkable variation in size and nucleotide composition
Source: Microb Genom. 2021 Dec 10;7(12):000720. doi: 10.1099/mgen.0.000720 (PMC8767329; doi:10.1099/mgen.0.000720)
Supplement: Supplementary material 1 [file mgen-7-0720-s001.pdf]

Title: **Characterization of the mitochondrial genomes of three powdery mildew pathogens reveals remarkable variation in size and nucleotide composition.**

Running title: Comparative mitochondrial genomics of powdery mildew fungi.

Authors/Affiliations: Alex Z. Zaccaron[1] and Ioannis Stergiopoulos[1*]

[1] Department of Plant Pathology, University of California Davis, Davis, CA, USA.

*Correspondence: Ioannis Stergiopoulos: University of California Davis, Department of Plant Pathology, One Shield Avenue, Davis, CA 95616-8751, USA, Tel: +1-530-400-9802, email: istergiopoulos@ucdavis.edu

Supplementary Tables

Table S1. Statistics of fungal mitochondrial (mt) genomes found in the NCBI Nucleotide and Organelle databases as of May 6, 2021. Mt genomes with no release date were not present in the NCBI Organelle database, but were obtained from the NCBI Nucleotide database. Genomes were sorted by length. Powdery mildew pathogens are highlighted. Columns 11 to 15 show the respective organism's taxonomy classification. The last column shows the percentage of the respective mt genome that are GC-rich (GC>50%) and calculated using a non-overlapping sliding window of 200 bp. Data for the mt genome of *Erysiphe necator* were obtained from Zaccaron et al (2021) [28].

Table S2. Short tandem repeats within the mitochondrial (mt) genomes of the powdery mildews *Blumeria graminis* f. sp. *tritici*, *Erysiphe pisi*, *Golovinomyces cichoracearum*, and *Erysiphe necator*. Tandem repeats were identified with the Tandem Repeat Finder program v4.09. Data for the mt genome of *E. necator* were obtained from Zaccaron et al (2021) [28].

Table S3. Classification of introns in the mitochondrial (mt) genomes of the powdery mildew pathogens *Blumeria graminis* f. sp. *tritici*, *Erysiphe pisi*, *Golovinomyces cichoracearum*, and *Erysiphe necator*. Information of intronic ORFs encoding homing endonucleases or reverse transcriptase is shown. Start and end coordinates are relative to the respective mt genome. Intron IDs are composed of the gene name that they are from followed by the intron number from 5' to 3' end. Data for the mtl genome of *E. necator* were obtained from Zaccaron et al (2021) [28].

Table S4. Core mitochondrial genes of powdery mildew species vary greatly in size and intron content. Shown are the total size (from start to stop codons) of each gene, the size of the mature transcripts (introns spliced out), the number of introns, and the total size of introns. Data for the mitochondrial genome of *Erysiphe necator* were obtained from Zaccaron et al (2021) [28].

Table S5. Mitochondrial (mt) introns are overall poorly conserved at the nucleotide level among the powdery mildew pathogens *Blumeria graminis* f. sp. *tritici* (Bg), *Erysiphe necator* (En), *Erysiphe pisi* (Ep), and *Golovinomyces cichoracearum* (Gc). The table shows pairwise identity values (%) of introns inserted into the same site between two species. If an intron is absent in one or both species, the corresponding identity value is denoted as NA. Introns were aligned globally using the Needleman-Wunsch algorithm implemented in the R package Biostrings. Identity values were calculated with the formula 100 * (identical positions)/(aligned positions + internal gap positions). Data for the mt genome of *E. necator* were obtained from Zaccaron et al (2021) [28].

Table S6. GC-rich regions in the mitochondrial (mt) genomes of *Blumeria graminis* f. sp. *tritici* and *Golovinomyces cichoracearum* are poorly conserved in other species. Queries correspond to GC-rich (GC%>50) segments within the mt genomes of *B. graminis* f. sp. *tritici* (bgt) and *G. cichoracearum* (gcic). Homology searches were performed with BLASTn against the nucleotide nr database (NCBI) with an e-value < 1e-5. From the 69 GC-rich regions of *B. graminis* f. sp. *tritici*, 58 matched only to a sequence (accession LR026995.1) that contains the mt genome of *B. graminis* f. sp. *tritici*, and was assembled along with its nuclear genome. From the 230 GC-rich regions of *G. cichoracearum*, 212 matched scaffolds from the genome assembly of the Oomycete *Albugo laibachii*.

Table S7. Features and statistics of the cytochrome *b* (*cob*) gene from four powdery mildew pathogens. Data for the mitochondrial genome of *Erysiphe necator* were obtained from Zaccaron et al (2021) [28].

Table S8. Summary of statistics, features, and accession numbers of cytochrome *b* (*cob*) genes from different fungal species. Members of the Leotiomycetes are shown at the top and powdery mildew pathogens are highlighted. For each species, NCBI accession numbers for the mitochondrial (mt) genome

and *cob* gene are shown, followed by the size of the *cob* coding sequence, coordinate of the exons, number of introns, *cob* start and end coordinates, total size of *cob*, average number of introns per kb of *cob* coding sequence, taxID of the species, description of the mt genome shown at NCBI, mt genome length, taxonomic lineage and genetic code number.

Table S9. Fungal mitochondrial (mt) genomes containing cytochrome *b* (*cob*) genes harboring reverse transcriptase (RT)-encoding ORFs within its introns. RT-encoding ORFs are embedded within introns inserted into nine sites of *cob* coding sequence, i.e., 99 (cob-99), 159 (cob-159), 247 (cob-212), 277 (cob-277), 311 (cob-311), 358 (cob-358), 541 (cob-541), and 687 (cob-687), using *cob* from *Erysiphe necator* as reference. For each fungal mt genome in the table, presence of an RT-encoding ORF within the respective intron insertion site is indicated with the coordinates of the respective ORF (5' to 3'). Absence of a RT-encoding ORF is indicated with black cells. Coordinates of the ORFs are in reference to the respective mt genome, for which the GenBank accession number is given in the third column.

Supplementary Figures

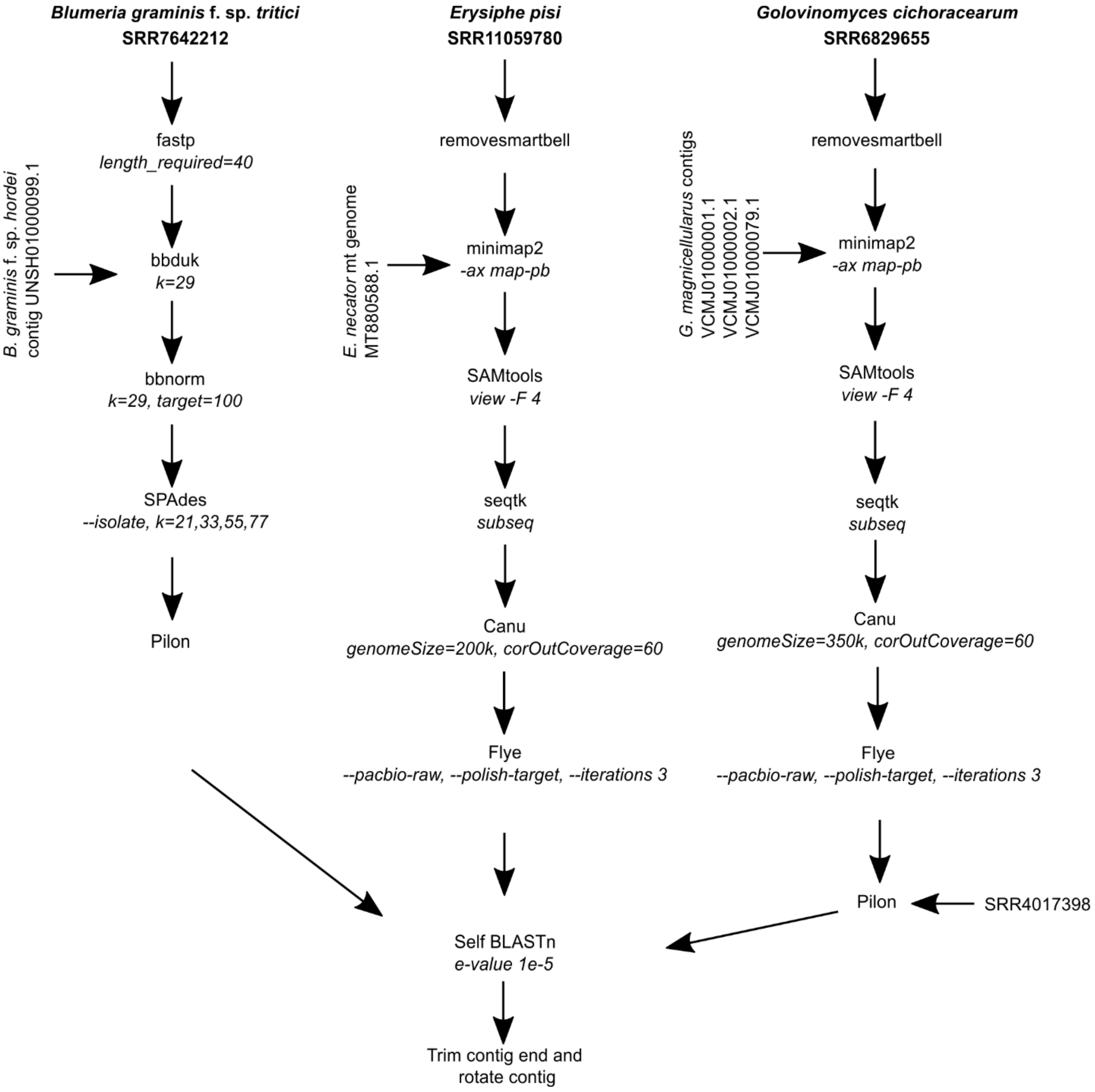

Fig. S1. Assembly workflow of the mitochondrial genomes of *Blumeria graminis* f. sp. *tritici*, *Erysiphe pisi*, and *Golovinomyces cichoracearum*. Arrows indicate flow of the tasks. Name of the software and parameters used are indicated. Accession numbers of assembled contigs and sequencing reads used are shown. Details are given in the Materials and Methods.

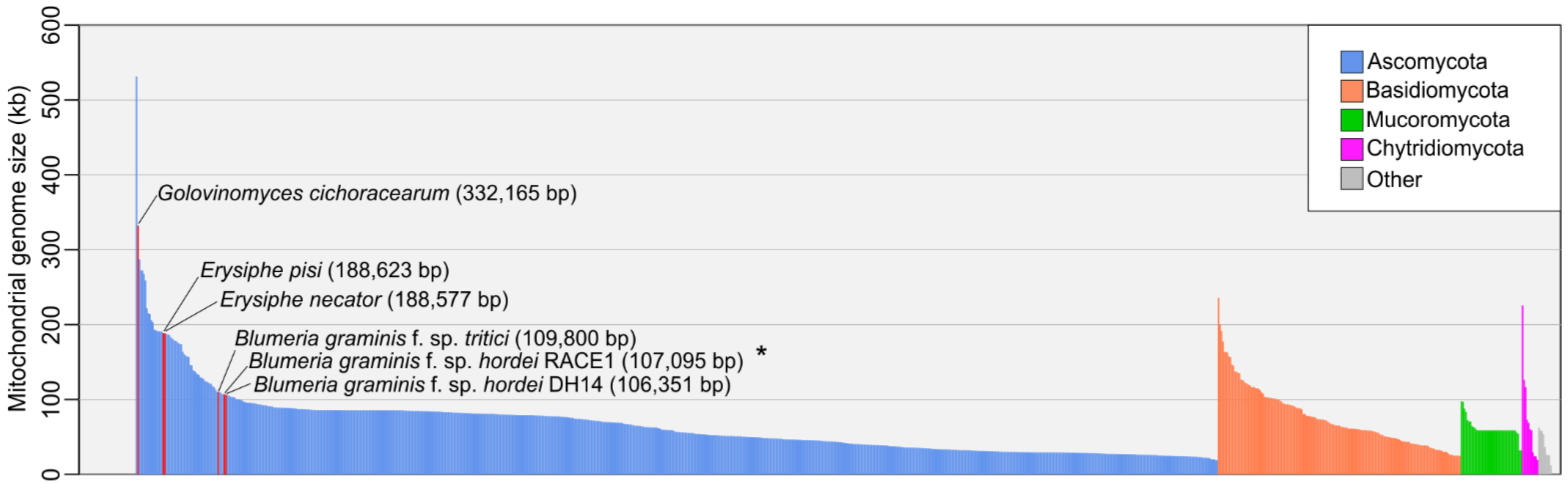

Fig. S2. Mitochondrial (mt) genome size comparison of 950 fungal mt genomes. Each mt genome is represented by a single bar in the graph and is color-coded based on the phylum of the fungal species that it is from. Blue: Ascomycota; orange: Basidiomycota; green: Mucoromycota; purple: Chytridiomycota; grey: Zoopagomycota, Blastocladiomycota, and Cryptomycota. The bars representing the mt genomes of the powdery mildew fungi *Blumeria graminis* f. sp. *tritici*, *B. graminis* f. sp. *hordei*, *Erysiphe necator, Erysiphe pisi,* and *Golovinomyces cichoracearum* are highlighted in red, even though they are all species of Ascomycota. The largest mt genome is that of *Morchella crassipes* (531.2 kb), whereas *Golovinomyces cichoracearum* has the second largest mt genome (332.2 kb). The complete list of the fungal species included in this bar graph can be found in Table S1. The size of the mt genome of *B. graminis* f. sp. *hordei* RACE1 marked with an asterisk is shown after trimming one of the 32,179 bp overlapping ends of the original assembled contig (139,274 bp).

(a)

```
Enec_atp9      1        TTAGCTACAACGGGTTTAATTGCAGGCGATATAGGAGTAGTTTTCGCAGCATTAATATTA  60
                        ||||||||||||||||||||||||||||||||||||||||||||||| |||||||||| |
Episi_mtgenome 145062   TTAGCTACAACGGGTTTAATTGCAGGCGATATAGGAGTAGTTTTCGCTGCATTAATATAA  145121

Enec_atp9      61       GGTGTAGCAATAAATCCTTCTTTAATAAGCCAATTATTCTCTTACGCTATACTTTGTTTT  120
                        ||||||||||||||| ||| ||||||||||||||||||||||||||||||||||||| ||
Episi_mtgenome 145122   GGTGTAGCAATAAATTCTTGTTTAATAAGCCAATTATTCTCTTACGCTATACTTTGTATT  145181

Enec_atp9      121      GCTTTTTGCATAAGAAACAGGATTATTTGCATTAATGATGGTCTTTTATATGTGGCTTAG  180
                        ||||||||||||| ||||||||||||||||||||||||||| ||||||||||||||||||
Episi_mtgenome 145182   GCTTTTTGCATAATAAACAGGATTATTTGCATTAATGATGGCCTTTTATATGTGGCTTAG  145241
```

(b)

```
Enec_atp9      2        TAGCTACAACGGGTTTAATTGCAGGCGATATAGGAGTAGTTTTCGCAGCATTAATATTAG  61
                        |||| ||||   ||||||||||||| | ||||||| | |||||||| |||||||||||||
Gcic_mtgenome  214637   TAGCCACAA---GTTTAATTGCAGG-GCTATAGGACTTGTTTTCGCTGCATTAATATTAG  214692

Enec_atp9      62       GTGTAGCAATAAATCCTTCTTTAATAAGCCAATTATTCTCTTACGCTATACTTTGTTTTG  121
                        |||||||||   |||||||||||| |||||||||||||||| |||||||||||| |
Gcic_mtgenome  214693   GTGTAGCAA---ATCCTTCTTTAAGAAGCCAATTATTCTCTAACGCTATACTTTTT----  214745

Enec_atp9      122      CTTTTTGCATAAGAAACAGGATTATTTGCATTAATGATGG-----TCTTTTATATGTGG  175
                         ||||||||   | ||||||||||||||||||||||||||     ||||||||||||||
Gcic_mtgenome  214746   -TTTTTGCACTTGCAACAGGATTATTTGCATTAATGATGGCTTTTTCTTTTATATGTGG  214803
```

Fig. S3: The mitochondrial (mt) genomes of the powdery mildew fungi *Erysiphe pisi* and *Golovinomyces cichoracearum* likely contain non-functional homologs of the *atp9* gene. Alignments obtained with BLASTn by querying the predicted coding sequence of the mt *atp9* gene of *E. necator* (QQY98148.1; 180 bp) against the mt genome of *B. graminis* f. sp. *tritici* (no hits; not shown), *E. pisi* (a), and *G. cichoracearum* (b). A premature stop codon in *E. pisi* and insertions/deletions in *G. cichoracearum* suggest that these two species contain non-functional *atp9* homologs, whereas *B. graminis* f. sp. *tritici* completely lost its *atp9* homolog. BLASTn search was performed with parameters *-evalue 1e-3* and *-task blastn*.

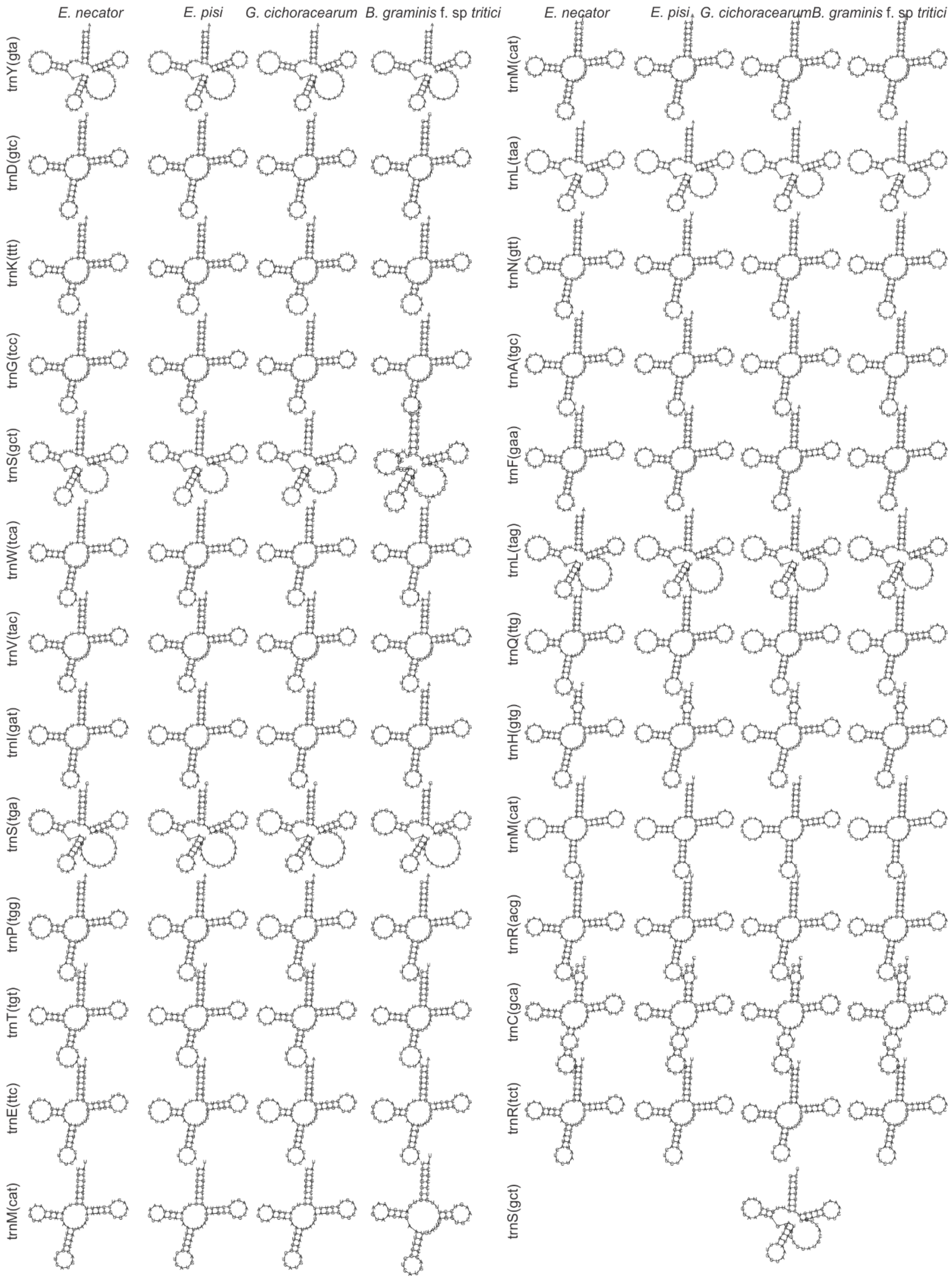
E. necator
E. pisi
G. cichoracearum
B. graminis f. sp tritici
E. necator
E. pisi
G. cichoracearum
B. graminis f. sp tritici
trnY(gta)
trnD(gtc)
trnK(ttt)
trnG(tcc)
trnS(gct)
trnW(tca)
trnV(tac)
trnI(gat)
trnS(tga)
trnP(tgg)
trnT(tgt)
trnE(ttc)
trnM(cat)
trnM(cat)
trnL(taa)
trnN(gtt)
trnA(tgc)
trnF(gaa)
trnL(tag)
trnQ(ttg)
trnH(gtg)
trnM(cat)
trnR(acg)
trnC(gca)
trnR(tct)
trnS(gct)

Fig. S4. Predicted secondary structures of mitochondrial (mt) tRNAs from the powdery mildew fungi *Blumeria graminis* f. sp. *tritici*, *Erysiphe necator*, *Erysiphe pisi*, and *Golovinomyces cichoracearum*. The mt-tRNAs are arranged from the top left to the bottom right of the figure according to their order in their respective mitochondrial genomes. Respective mt-tRNA-anticodons are shown between parentheses. Mt-tRNA and mt-tRNA-anticodon structures were predicted with MITOS2 web server (http://mitos2.bioinf.uni-leipzig.de/index.py). Data for the mitochondrial genome of *E. necator* were obtained from Zaccaron et al (2021) [28]

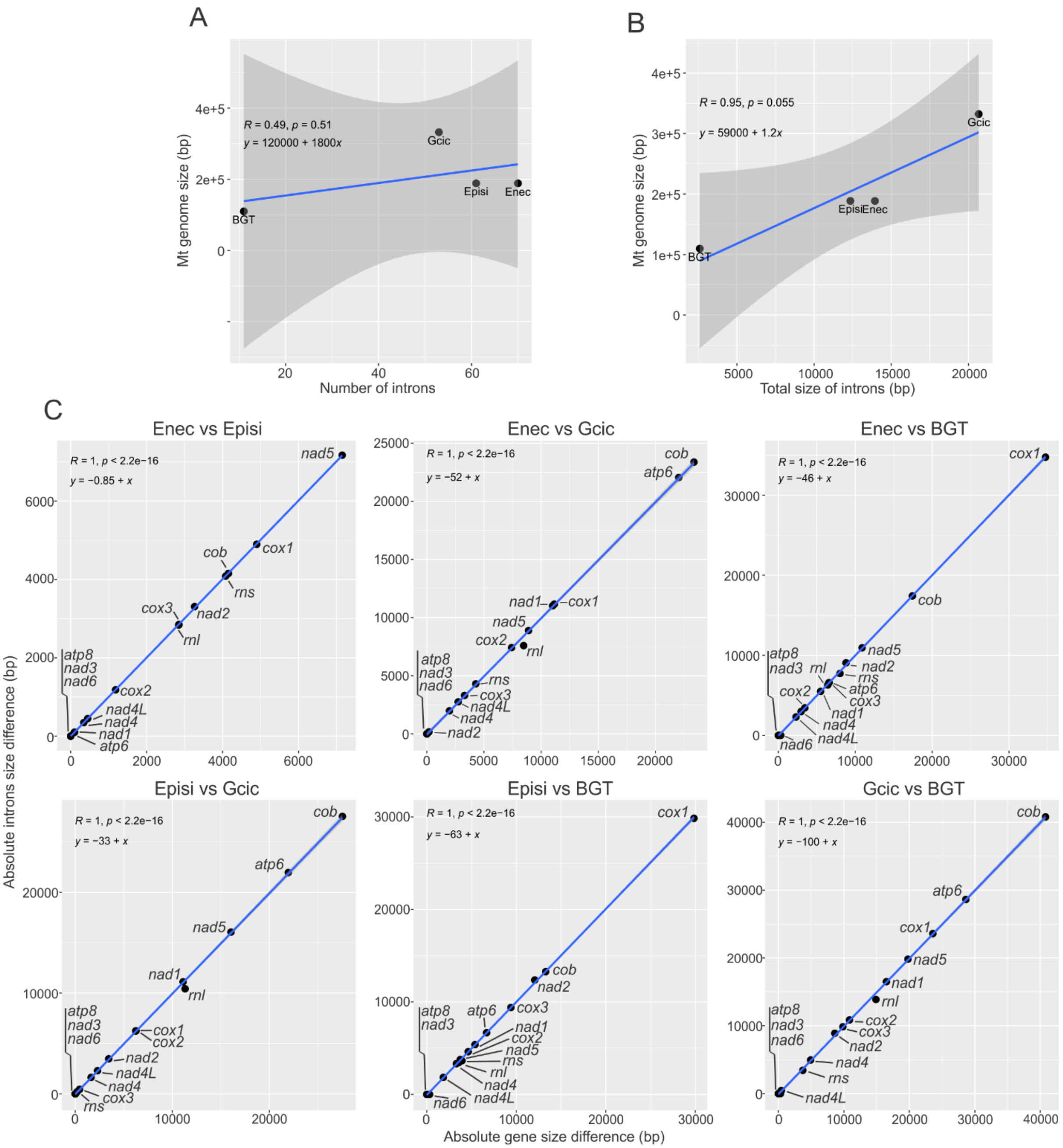

Fig. S5: Size of mitochondrial (mt) genomes and size of mt genes correlate with the size of intronic sequences among powdery mildew pathogens. (A) Scatter plot showing the weak correlation between the size of the mt genomes of *Blumeria graminis* f. sp. *tritici* (Bgt), *Erysiphe necator* (En), *E. pisi* (Ep), and *Golovinomyces cichoracearum* (Gc), and the number of introns present in them. (B) Scatter plot showing the strong correlation between the size of the mt genomes of Bgt, En, Ep and Gc and the total length of introns present in them. (C) Scatter plots showing the strong correlation between differences in gene and intron length for all core mt genes among pairwise comparisons of Bgt, En, Epi and Gc. The plots indicate that the differences in gene length among the four powdery mildew pathogens is explained by their differences in intron length. For all scatter plots in (A), (B) and (C), regression lines are shown in blue and were determined with the *geom_smooth* function from the R package *ggplot2*, utilizing the *lm* method. Dark areas represent confidence intervals (95%). Correlation coefficient, *p*-value and the equation of the regression line are shown at the top left corner of each plot. Data for the mt genome of *E. necator* were obtained from Zaccaron et al (2021) [28].

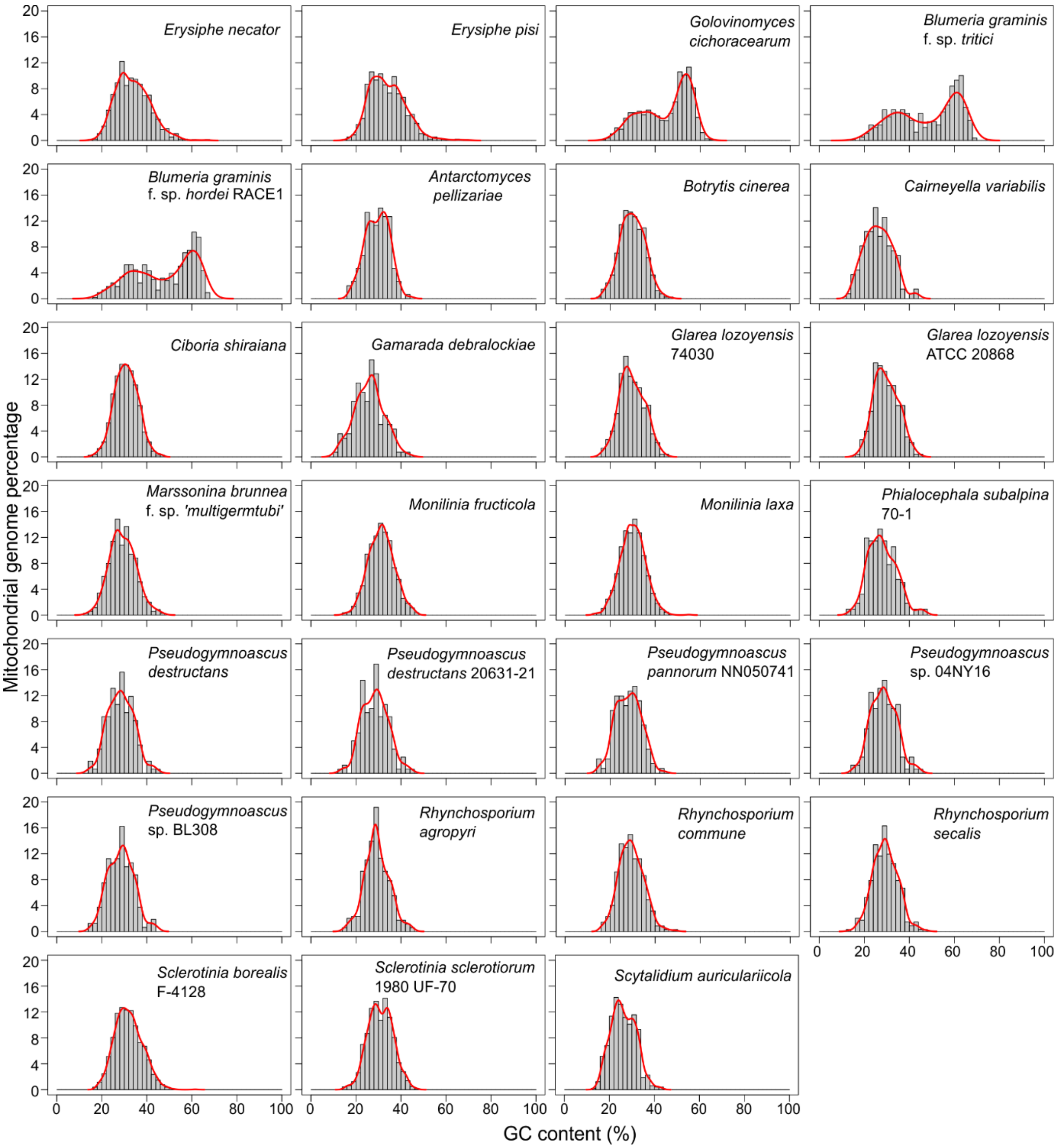

Fig. S6. GC content distribution in the mitochondrial (mt) genomes of members of Leotiomycetes. Histograms were generated by calculating the GC content within a non-overlapping sliding window of 200 bp across each respective mt genome. Solid lines represent the distributions estimated based on kernel density of the histograms. The graphs show that the mt genomes of the powdery mildew species *Golovinomyces cichoracearum*, *Blumeria graminis* f. sp. *tritici*, and *B. graminis* f. sp. *hordei* exhibit a bimodal distribution of GC content with two peaks at 36% and 53%, 35% and 61%, and 35% and 61%, respectively. In contrast, the mt genomes of *E. necator, E. pisi*, and of the other Leotiomycetes have a unimodal distribution of GC content. Accession numbers of the mt genomes are shown in Table S1. Data for the mitochondrial genome of *E. necator* were obtained from Zaccaron et al (2021) [28]. Percentage values for the mt genome of *B. graminis* f. sp. *hordei* RACE1 were obtained after trimming one of the 32,179 bp overlapping ends of the original assembled contig (139,274 bp). The nucleotide sequence of *B. graminis* f. sp. *hordei* isolate DH14 was not available at NCBI, Ensembl, or JGI MycoCosm at the time of publication of this work.

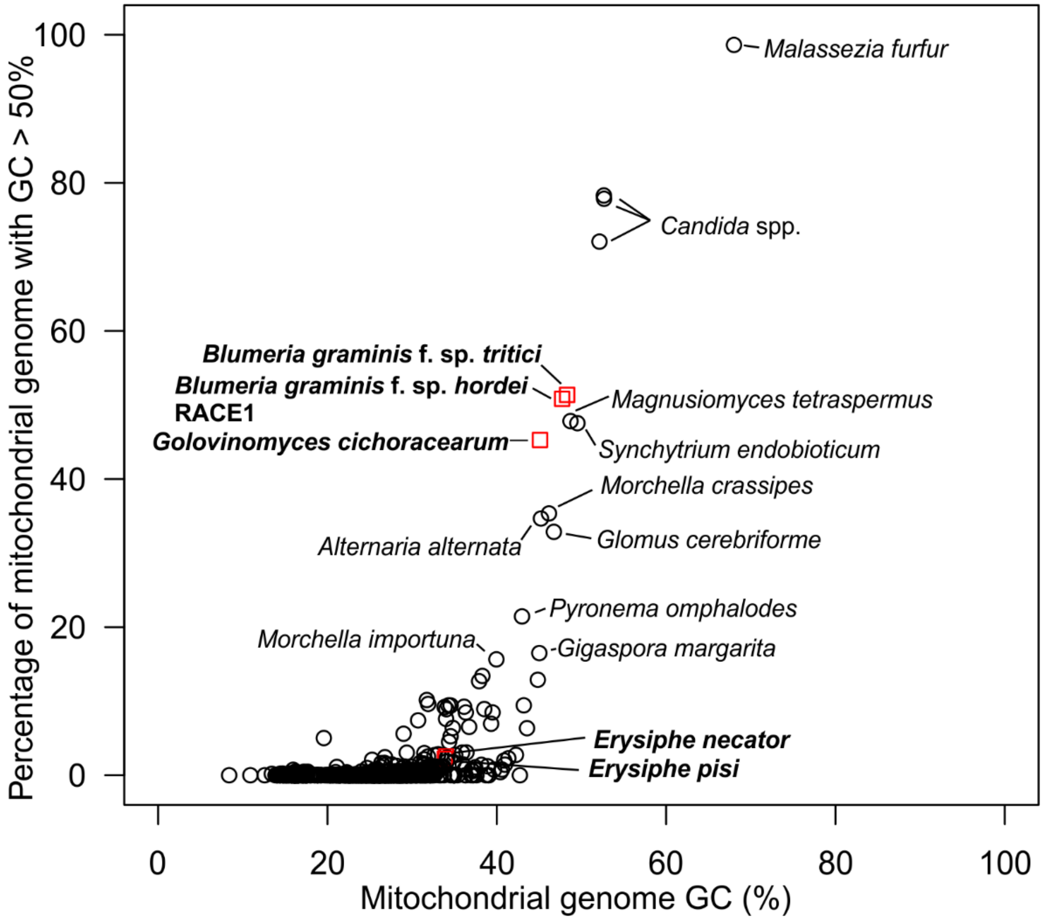

Fig. S7. GC content comparison of among 949 fungal mitochondrial (mt) genomes. Each circle in the scatterplot represents a mt genome, plotted on the X-axis based on its overall GC content and on the Y-axis based on what percentage of it has a GC content >50%. The mt genomes of the five powdery mildew species are shown as red squares. The scatterplot shows that the mt genomes of *Blumeria graminis* f. sp. *tritici* (GC = 48%; $GC_{>50\%}$: 52%), %), *Blumeria graminis* f. sp. *hordei* (GC = 47.7%; $GC_{>50\%}$: 50%), and *Golovinomyces cichoracearum* (GC = 45%; $GC_{>50\%}$: 46%) have from the highest percentages of GC content among all other fungal mt genomes. In contrast, *Erysiphe necator* (GC = 55%; $GC_{>50\%}$: 3%), and *E. pisi* (GC = 33%; $GC_{>50\%}$: 4%) have mt genomes with an average GC content. The fungal species represented in this graph are listed in Table S1. Percentages of the mt genomes with GC > 50% were determined using a nonoverlapping sliding window of 200 bp. Percentage values for the mt genome of *B. graminis* f. sp. *hordei* RACE1 were obtained after trimming one of the 32,179 bp overlapping ends of the original assembled contig (139,274 bp). The nucleotide sequence of *B. graminis* f. sp. *hordei* isolate DH14 was not available at NCBI, Ensembl, or JGI MycoCosm at the time of publication of this work.

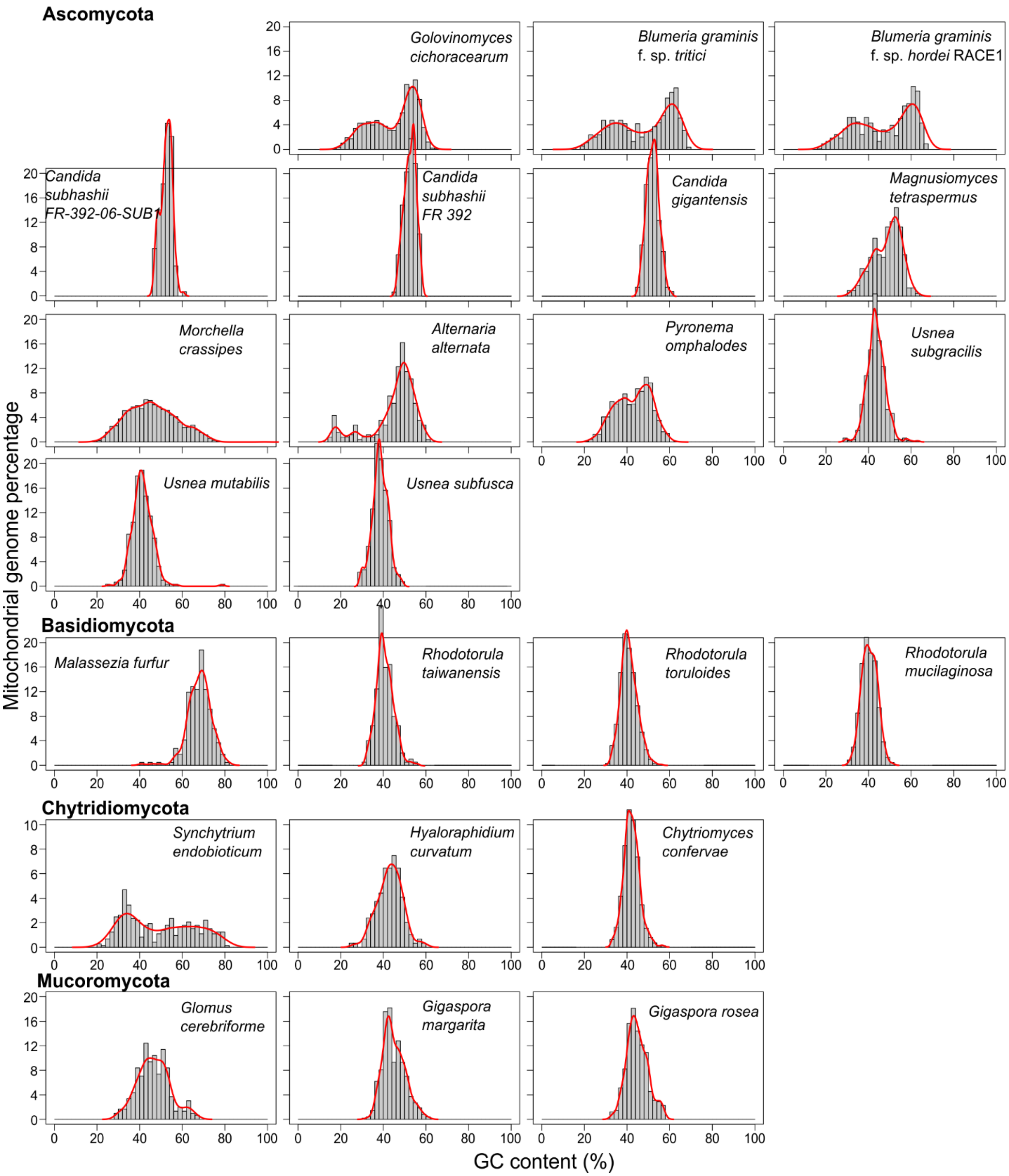

Fig. S8. GC content distribution in fungal mitochondrial (mt) genomes with an overall GC content higher than 40%. Histograms were generated by calculating the GC content within a non-overlapping sliding window of 200 bp across each respective mt genome. Solid lines represent the distributions estimated based on the kernel density of the histograms. The graphs show that the mt genomes of the powdery mildew species *Golovinomyces cichoracearum* and *Blumeria graminis* f. sp. *tritici,* and *B. graminis* f. sp. *hordei* (shown at the top) exhibit a bimodal distribution of GC content with two peaks at 36 and 53%, 35% and 61%, and 35 and 61%, respectively. A similar pattern of GC content distribution although less pronounced is seen in the mt genomes of *Magnusiomyces tetraspermus, Morchella crassipes, Alternaria*

alternata, Pyronema ompalodes, Synchytrium endobioticum, and *Glomus cerebriforme.* All other mt genomes with a GC content of higher than 40% have a unimodal distribution of GC content. Accession numbers of the mt genomes are shown in Table S1. Percentage values for the mt genome of *B. graminis* f. sp. *hordei* RACE1 were obtained after trimming one of the 32,179 bp overlapping ends of the original assembled contig (139,274 bp). The nucleotide sequence of *B. graminis* f. sp. *hordei* isolate DH14 was not available at NCBI, Ensembl, or JGI MycoCosm at the time of publication of this work.
